# Mutations in six nephrosis genes delineate a pathogenic pathway amenable to treatment

**DOI:** 10.1038/s41467-018-04193-w

**Published:** 2018-05-17

**Authors:** Shazia Ashraf, Hiroki Kudo, Jia Rao, Atsuo Kikuchi, Eugen Widmeier, Jennifer A. Lawson, Weizhen Tan, Tobias Hermle, Jillian K. Warejko, Shirlee Shril, Merlin Airik, Tilman Jobst-Schwan, Svjetlana Lovric, Daniela A. Braun, Heon Yung Gee, David Schapiro, Amar J. Majmundar, Carolin E. Sadowski, Werner L. Pabst, Ankana Daga, Amelie T. van der Ven, Johanna M. Schmidt, Boon Chuan Low, Anjali Bansal Gupta, Brajendra K. Tripathi, Jenny Wong, Kirk Campbell, Kay Metcalfe, Denny Schanze, Tetsuya Niihori, Hiroshi Kaito, Kandai Nozu, Hiroyasu Tsukaguchi, Ryojiro Tanaka, Kiyoshi Hamahira, Yasuko Kobayashi, Takumi Takizawa, Ryo Funayama, Keiko Nakayama, Yoko Aoki, Naonori Kumagai, Kazumoto Iijima, Henry Fehrenbach, Jameela A. Kari, Sherif El Desoky, Sawsan Jalalah, Radovan Bogdanovic, Nataša Stajić, Hildegard Zappel, Assel Rakhmetova, Sharon-Rose Wassmer, Therese Jungraithmayr, Juergen Strehlau, Aravind Selvin Kumar, Arvind Bagga, Neveen A. Soliman, Shrikant M. Mane, Lewis Kaufman, Douglas R. Lowy, Mohamad A. Jairajpuri, Richard P. Lifton, York Pei, Martin Zenker, Shigeo Kure, Friedhelm Hildebrandt

**Affiliations:** 1 Department of Medicine, Boston Children’s Hospital, Harvard Medical School, Boston, MA USA; 2 Department of Biosciences, Jamia Millia Islamia, New Delhi, India; 3 Department of Pediatrics, Tohoku University School of Medicine, 1-1 Seiryo-machi, Aoba-ku, Sendai, Miyagi 980-8574 Japan; 4 Department of Pharmacology, Brain Korea 21 PLUS Project for Medical Sciences, Yonsei University College of Medicine, Seoul, 03722 Korea; 5 Department of Biological Sciences, National University of Singapore, Singapore, Singapore; 6 Mechanobiology Institute, National University of Singapore, Singapore, Singapore; 7 Laboratory of Cellular Oncology, Center for Cancer Research, National Cancer Institute, National Institutes of Health, Bethesda, MD USA; 8 Division of Nephrology, Icahn School of Medicine at Mount Sinai, New York, NY USA; 9 Manchester Centre for Genomic Medicine, St Mary’s Hospital, Central Manchester University Hospitals NHS Foundation Trust, Manchester Academic Health Science Centre, Manchester, UK; 10 Institute of Human Genetics, University Hospital Magdeburg, Magdeburg, Germany; 11 Department of Medical Genetics, Tohoku University School of Medicine, 1-1 Seiryo-machi, Aoba-ku, Sendai, Miyagi 980-8574 Japan; 12 Department of Pediatrics, Kobe University Graduate School of Medicine, 7-5-2 Kusunoki-cho, Chuo-ku, Kobe, 650-0017 Japan; 13 2nd Department of Internal Medicine, Kansai Medical University, 2-3-1 Shin-machi, Hirakata-shi, Osaka, 573-1191 Japan; 14 Department of Nephrology, Hyogo Prefectural Kobe Children’s Hospital, 1-6-7 Minatojima-minamimachi, Chuo-ku, Kobe, Hyogo 650-0047 Japan; 15 Department of Pediatrics, Himeji Red Cross Hospital, 1-12-1 Shimoteno, Himeji, Hyogo 670-8540 Japan; 16 Department of Pediatrics, Gunma University Graduate School of Medicine, 3-39-22 Showa-machi, Maebashi, Gunma 371-8511 Japan; 17 Academic Renal Unit, School of Clinical Science, University of Bristol, Dorothy Hodgkin Building, Whitson Street, Bristol, BS1 3NY United Kingdom; 18 Division of Cell Proliferation, United Centers for Advanced Research and Translational Medicine, Tohoku University Graduate School of Medicine, Sendai, Miyagi 980-8575 Japan; 19 Department of Pediatric Nephrology, Children’s Hospital, Memmingen, Germany; 20 Pediatric Nephrology Center of Excellence and Pediatric Department, King Abdulaziz University, Jeddah, Saudi Arabia; 21 Pathology Department, King Abdulaziz University, Jeddah, Saudi Arabia; 22 Institute for Mother and Child Health Care of Serbia “Dr Vukan Čupić”, Department of Nephrology, University of Belgrade, Faculty of Medicine, Belgrade, 11000 Serbia; 23 Department for Paediatrics II, University of Göttingen, Göttingen, Germany; 24 Department of Nephrology, Asfendiyarov Kazakh National Medical University, Almaty, Kazakhstan; 25 Department of Nephrology, Luzerner Kantonsspital, Lucerne, Switzerland; 26 Department of Pediatrics, University Medical Center Innsbruck, Innsbruck, Austria; 27 Department of Pediatric Nephrology, Hannover Medical School, Hannover, Germany; 28 Department of Pediatric Nephrology and Medical Genetics, Institute of Child Health and Hospital for Children, TN Dr.M.G.R. Medical University, Chennai, India; 29 Division of Pediatric Nephrology, Department of Pediatrics, All India Institute of Medical Sciences, New Delhi, India; 30 Department of Pediatrics, Center of Pediatric Nephrology & Transplantation, Kasr Al Ainy School of Medicine, Cairo University, Cairo, Egypt; 31 Department of Genetics, Yale University School of Medicine, New Haven, CT 06510 USA; 32 Laboratory of Human Genetics and Genomics, The Rockefeller University, New York, NY 10065 USA; 33 Division of Nephrology, University Health Network, and University of Toronto, Toronto, ON Canada

## Abstract

No efficient treatment exists for nephrotic syndrome (NS), a frequent cause of chronic kidney disease. Here we show mutations in six different genes (*MAGI2, TNS2, DLC1, CDK20, ITSN1, ITSN2*) as causing NS in 17 families with partially treatment-sensitive NS (pTSNS). These proteins interact and we delineate their roles in Rho-like small GTPase (RLSG) activity, and demonstrate deficiency for mutants of pTSNS patients. We find that CDK20 regulates DLC1. Knockdown of *MAGI2*, *DLC1*, or *CDK20* in cultured podocytes reduces migration rate. Treatment with dexamethasone abolishes RhoA activation by knockdown of *DLC1* or *CDK20* indicating that steroid treatment in patients with pTSNS and mutations in these genes is mediated by this RLSG module. Furthermore, we discover *ITSN1* and *ITSN2* as podocytic guanine nucleotide exchange factors for Cdc42. We generate *Itsn2*-*L* knockout mice that recapitulate the mild NS phenotype. We, thus, define a functional network of RhoA regulation, thereby revealing potential therapeutic targets.

## Abstract (web summary)

Nephrotic syndrome is the second most common chronic kidney disease but there are no targeted treatment strategies available. Here the authors identify mutations of six genes codifying for proteins involved in cytoskeleton remodelling and modulation of small GTPases in 17 families with nephrotic syndrome.

## Introduction

Nephrotic syndrome (NS) is the second most frequent cause of chronic kidney disease in the first three decades of life, requiring dialysis or transplantation for survival^1^. Based on its response to steroid treatment, steroid sensitive NS (SSNS) is distinguished from steroid resistant NS (SRNS). No efficient treatment for SRNS exists, and very little is known about disease mechanisms. Mutations in more than 40 genes have been identified as causing monogenic (single-gene) forms of NS. Interestingly, most of the encoded gene products localize to renal glomerular podocytes, confirming that podocyte loss of function is a critical part of the pathogenesis of NS and that any and all of these proteins are important for the maintenance of glomerular function^2,3^. These findings have thereby helped define protein interaction complexes and functional pathways that could be targeted for future potential treatment of NS^2,4–7^. In addition, the mechanistic causes of steroid resistance have been a conundrum for almost six decades of their use. So far, only one causative gene (*EMP2*) has been discovered in SSNS^8^. We recently demonstrated in a large cohort of 1780 families with SRNS that in about 30% of individuals with SRNS a monogenic cause can be detected, indicating that additional single gene causes of NS must exist that are yet to be identified^9^.

Therefore by performing whole exome sequencing in individuals with partially treatment sensitive NS (pTSNS), we discover six novel monogenic causes for pTSNS. Strikingly, all six encoded proteins interacted within a functional module of podocytic regulation of Rho-like small GTPase (RLSG) and are at the intersection between steroid sensitivity vs. steroid resistance of NS.

## Results

### Mutations in six novel genes cause NS in humans

To identify additional genes mutated in NS we performed homozygosity mapping (HM)^10^ combined with whole exome sequencing (WES)^11^ in multiple families with NS. We also performed high-throughput exon sequencing^12,13^ in a worldwide cohort of ~1000 additional families with NS, examining specific candidate genes for NS based on genetic mouse models of NS.

**Fig. 1:**
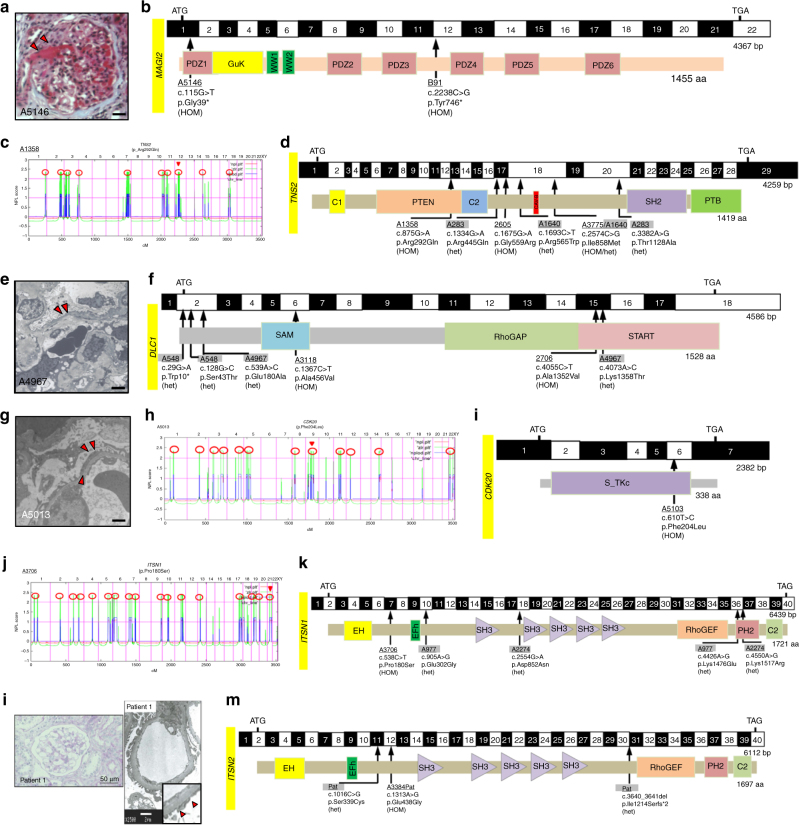
High-throughput sequencing reveals recessive mutations of *MAGI2*, *TNS2, DLC1, CDK20, ITSN1,* or *ITSN2* as causing NS in humans. **a** Renal histology of individual A5146-21 with focal segmental glomerulosclerosis (FSGS) and MAGI2 mutation (scale bar = 50 μm). **b** Exon structure of human *MAGI2* cDNA and mutations. Below is the protein domain structure of MAGI2, showing GuK, WW1, WW2, and six different PDZ domains. Two different homozygous truncating mutations of *MAGI2* were detected in two families with NS and neurological impairment. **c** Homozygosity mapping identifies ten recessive candidate loci (red circles) in family A1358 with NS, and WES identifies a homozygous mutation of *TNS2* (p.Arg292Gln). Non-parametric lod scores (NPL) were calculated and plotted across the human genome. The *TNS2* locus (arrowhead) is positioned within one of the maximum NPL peaks on chromosome 12q. **d** Exon and protein domain structure of human TNS2. Six different *TNS2* mutations were detected in five families with NS. Family numbers and amino acid changes are given (Supplementary Table 1). Arrow heads denote altered nucleotides. Lines and arrows indicate positions of mutations in relation to exons and protein domains. Family numbers with compound heterozygous mutations (het) are highlighted in gray. **e** Renal histology of individual A4967-21 with FSGS and *DLC1* mutations. TEM reveals podocyte foot process effacement (arrow heads, magnification 8000x). **f** Exon and protein domain structure of human DLC1. The SAM, RhoGAP, and START domains are depicted by colored bars, in relation to encoding exon position. Six different *DLC1* mutations were detected in four families with NS. Positions of amino acid changes (Supplementary Table 1) are marked with arrowheads. **g** Renal histology of individual A5013-21 with membranoproliferative glomerulonephritis (MPGN) and mutation in *CDK20*. TEM reveals podocyte foot process effacement and thickening of glomerular basement membrane (arrow heads, magnification 7000x). **h** Homozygosity mapping identifies twelve recessive candidate loci (red circles) in family A5013 with NS, and WES identifies a homozygous mutation of *CDK20* (p.Phe204Leu), positioned within one of the maximum NPL peaks on chromosome 9q. **i** Exon and protein domain structure of human CDK20. The serine threonine kinase (S_TKc) domain is depicted by a colored bar, in relation to encoding exon position. One homozygous mutation in *CDK20* was detected in family A5013 with NS. **j** Homozygosity mapping in family A3706 with NS identifies 17 recessive candidate loci (red circles), and WES identifies a homozygous mutation of *ITSN1* (p.Pro180Ser), positioned within one of the maximum NPL peaks on chromosome 21q. **k** Exon and protein domain structure of human ITSN1. Five different *ITSN1* mutations were detected in three families with NS. **l** Renal histology of Patient-1 with mutations in *ITSN2* revealed minimal change disease (scale bar = 50 μm). EM showed foot process effacement (scale bar = 2 μm). **m **Exon and protein domain structure of human ITSN2. Three different *ITSN2* mutations were detected in two families with NS

**Fig. 2:**
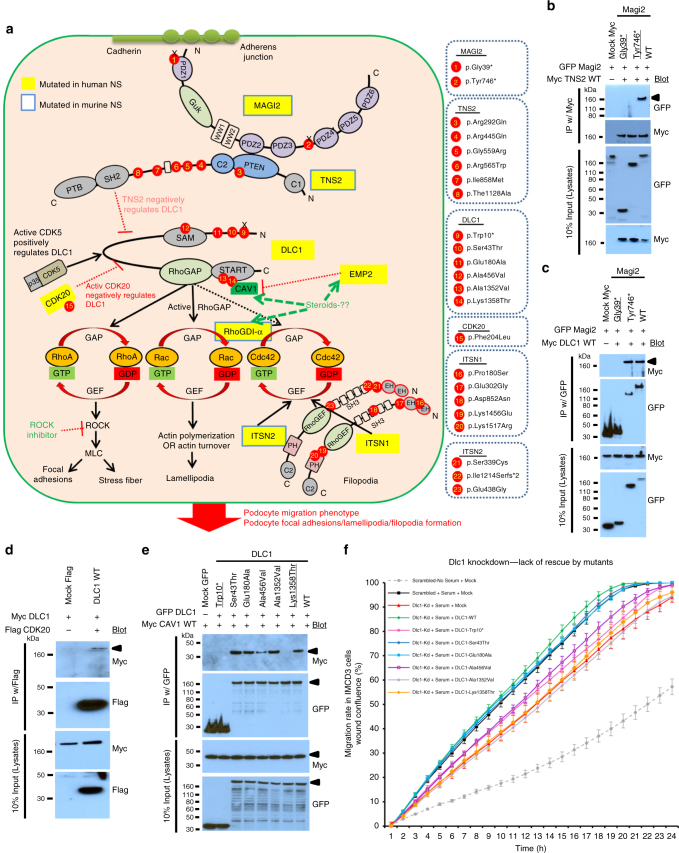
Gene products of *MAGI2*, *TNS2*, *DLC1*, *CDK20*, and *CAV1* physically and functionally interact to regulate RhoA/Rac1/Cdc42 activation. **a** Identification of six novel monogenic causes of NS reveals a regulatory network of RhoA activation. The large rounded square symbolizes a podocyte. All six proteins MAGI2, TNS2, DLC1, CDK20, ITSN1, and ITSN2, in which recessive defects were detected herein as novel causes of pTSNS, interact physically or functionally to regulate RhoA/Rac1/Cdc42. Yellow labels highlight proteins encoded by genes, which if mutated give rise to monogenic nephrosis as shown in this study (MAGI2, TNS2, DLC1, CDK20, ITSN1, and ITSN2) or as published (EMP2, ARHGDIA). Blue frame around yellow labels indicate that there is also a monogenic mouse model of NS or a zebrafish model known, as shown in this study for ITSN2 or as published for MAGI2, TNS2, EMP2, and RhoGDI-α. For each of the proteins, MAGI2, TNS2, DLC1, CDK20, TLN1, or ITSN1, the protein domains are shown. Red circles denote positions of mutations that we found in patients. Truncation mutations are represented by “x”. By identifying novel monogenic causes of NS we discovered a cluster proteins that regulate Rho/Rac/Cdc42 activation as being central for the pathogenesis of these patients. **b** MAGI2 interacts with TNS2 upon co-overexpression and coimmunoprecipitation (coIP) in HEK293T cells. Both mutant MAGI2 clones, Gly39* and Tyr746* (underlined) that reflect alleles of NS patients A5146-21 and B91 respectively, abrogate this interaction. **c** MAGI2 interacts with DLC1 upon co-overexpression and coIP in HEK293T cells. One mutant MAGI2 clone Gly39* (underlined) reflecting a mutation of NS patient A5146-21 abrogates this interaction. **d** DLC1 interacts with CDK20 upon co-overexpression in HEK293T cells. **e** DLC1 interacts with CAV1. Two mutant DLC1 c-DNA clones, reflecting Trp10* and Lys1358Thr alleles of NS patients A548-21 and A4967-21 respectively, lack this interaction. **f** In IMCD3 cells, migration rate is induced in the presence of serum as compared to scrambled control. Knockdown of *Dlc1* in IMCD3 cells using mouse *Dlc1* siRNA #1 impairs cell migration rate (red vs. black curve with serum). The decrease in migration is rescued by transfection with full-length human DLC1 cDNA (green curve). Transfection with four out ot six mutants (Trp10*, Ala456Val, Ala1352Val, and Lys1358Thr) failed to rescue this migratory phenotype

Mice lacking *Magi2* develop NS^14–16^. We sequenced all *MAGI2* exons in 400 patients with NS. We identified homozygous truncating mutations in the *MAGI2* gene (p.Gly39* and p.Tyr746*) in two individuals with SRNS and neurologic impairment (Fig. 1a, b, Supplementary Table 1, Supplementary Fig. 1A). p.Gly39* was detected in an affected individual of Arab descent. p.Tyr746* was due to maternal isodisomy for chromosome 7. We thereby discovered recessive *MAGI2* mutations as a cause of SRNS with neurologic involvement in humans (Fig. 2a). Very recently, recessive mutations in *MAGI2* in humans with NS have been confirmed^17^.

In individual A1358 of Turkish origin with early-onset NS and histology of minimal change nephrotic syndrome (MCNS) (Supplementary Table 1), HM yielded ten regions of homozygosity by descent, none of which coincided with any of the known recessive SRNS loci (Fig. 1c). By WES, we detected a novel homozygous missense mutation (p. Arg292Gln) in the TNS2 (*tensin-2*) gene on chromosome 12q13.13 (Supplementary Table 2). The mutation alters an amino acid residue conserved throughout evolution from *C. intestinalis* on. Direct inspection of sequence alignments did not yield a mutation in any of the 30 known SRNS genes^3^, and no additional homozygous truncating mutations were detected in any other gene within the mapped candidate regions. By high-throughput exon sequencing, we detected in 4 additional families (A283, 2605, A3775, and A1640) 5 additional homozygous or compound heterozygous missense mutations of *TNS2* (Fig. 1d, Supplementary Table 1, Supplementary Fig. 1B). The variant p.Ile858Met apparently represents a founder allele from India (Supplementary Table 1). Altered amino acid residues were well conserved throughout evolution (Supplementary Table 1). We thereby discovered recessive *TNS2* mutations as a novel cause of NS (Fig. 2a). We introduce the term “NPHS19” for this distinct entity of NS.

Because TNS2 protein is known to interact with DLC1 (deleted in liver cancer 1)^18,19^, we hypothesized that mutations of *DLC1* may also cause NS. By high-throughput exon sequencing, we identified recessive mutations of *DLC1* in 4 families with NS (Supplementary Table 1). Specifically, in individual A548-21 with FSGS, we detected two compound heterozygous mutations: p.Trp10* and p.Ser43Thr. In an individual of Arab descent (A4967-21) with SRNS and a biopsy showing focal segmental glomerulosclerosis (FSGS) (Fig. 1e), we identified two compound heterozygous mutations, p.Glu180Ala and p.Lys1358Thr, in *DLC1* (Fig. 1f, Supplementary Table 1, Supplementary Fig. 1C). In an affected individual from Asia (A3118-21), we detected a homozygous mutation (p.Ala456Val) in exon 6 of *DLC1* with amino acid conservation since *C.elegans* (Fig. 1f, Supplementary Table 1, Supplementary Fig. 1C). In family 2706 we identified a homozygous missense mutation (p.Ala1352Val) in exon 17 of *DLC1* (Fig. 1f, Supplementary Table 1). We thereby discovered recessive *DLC1* mutations as a novel cause of pTSNS (Fig. 2a). We introduce the term “NPHS20” for this distinct entity of NS. Interestingly, a distinct disease phenotype of partially treatment sensitive nephrotic syndrome (pTSNS) occurred in four of the five individuals with recessive *TNS2* and in two of the four individuals with *DLC1* mutations. pTSNS that mostly manifested between 1 and 7 years of age, with the exception of A548-21 and 2706 where the onset of NS was in adulthood (Supplementary Table 1). Interestingly, these individuals showed complete or partial response to prednisolone or cyclosporine A.

In family A5013 of Arab origin, an individual had pTSNS with the unusual renal histology of membranoproliferative glomerulonephritis (MPGN) (Fig. 1g). HM yielded 12 regions of homozygosity by descent, none of which coincided with any of the known recessive NS loci (Fig. 1h). By WES, we detected a novel homozygous missense mutation (p.Phe204Leu) in exon 6 of the *CDK20* gene on chromosome 9q22.1, encoding “cyclin-dependent kinase 20” (Fig. 1i, Supplementary Table 1, Supplementary Table 2). The mutation alters an amino acid residue conserved throughout evolution from *C. elegans* on (Supplementary Fig. 1D).

By HM and WES in two siblings of Arab family A3706 (Fig. 1j, Supplementary Table 1, Supplementary Table 2) with early-onset NS we identified a homozygous missense mutation of *ITSN1* (Intersectin 1) at a highly conserved amino acid residue (p.Pro180Ser). By high-throughput sequencing, we identified two additional families (A977 and A2274) with four additional compound heterozygous missense mutations of *ITSN1* (Fig. 1k, Supplementary Table 1, Supplementary Fig. 1E). Although the mice lacking *Itsn1* have been shown to exhibit a neuronal phenotype^20^, none of individuals with early-onset NS identified here showed any brain abnormalities. We introduce the term “NPHS21” for this distinct entity of NS.

WES was also performed in five members of a Japanese family (two patients and their unaffected members) (Supplementary Fig. 2). Both affecteds in this family (Pat1, Pat 2) had pTSNS and renal histology in both the siblings showed MCNS (Fig. 1l). By WES, we identified two compound heterozygous mutations in trans in *ITSN2* (intersectin 2), a missense mutation (p.Ser339Cys) in exon 11 and a frame-shift deletion (p.Ile1214Serfs*2) in exon 30 of *ITSN2* (Fig. 1m, Supplementary Fig. 1F, Supplementary Table 1, Supplementary Table 3). Additionally, in an individual from Arab with pTSNS (A3384), we detected a homozygous missense mutation (p.Glu438Gly) in exon 12 of *ITSN2* (Fig. 1m, Supplementary Table 1). We thereby discovered recessive *ITSN2* mutations as novel cause of pTSNS (Fig. 2a). We introduce the term “NPHS22” for this distinct entity of NS. Interestingly, all three patients (from two families) with *ITSN2* mutations had pTSNS, as well as two out of four patients (from three families) with *ITSN1* mutations. Similar to patients with *TNS2*, *DLC1* or *CDK20* mutations, most patients with *ITSN2* or *ITSN1* mutations also showed partial or complete response to Cyclosporine A.

For all mutations in all six genes segregation status was compatible with recessive inheritance (Supplementary Table 1). Supplementary Table 4 lists all the variant frequencies for all the identified mutations in the Exome Aggregation Consortium (ExaC) and the genome aggregation database (GnomAD) along with their prediction scores using Mutation Taster (http://www.mutationtaster.org/) and Polyphen 2 (http://genetics.bwh.harvard.edu/pph2/) in silico tools. As a negative control, we performed screening for all variants that we detected in the pTSNS patients in an in-house cohort of exome data from 248 NPHP cases. This control did not reveal any homozygous carriers (Supplementary Table 4).

### Three of the six proteins interact with DLC1

We discovered that four proteins MAGI2, TNS2, DLC1, and CDK20, in which we detected recessive defects as novel causes of pTSNS, interact physically or functionally to regulate RhoA/Rac1/Cdc42. Specifically, we detected or confirmed the following protein–protein interactions:

First, because MAGI2 was known to interact with PTEN^21^, and because TNS2 contains a PTEN domain (Figs 1d and 2a), we hypothesized that MAGI2 may also interact with TNS2. By co-overexpression and co-immunoprecipitation (coIP) of wildtype and mutant cDNA clones in HEK293T cells, we found that MAGI2 in fact interacts with TNS2 (Fig. 2b), which we confirmed in a confirmatory coIP (Supplementary Fig. 3A). Both mutant MAGI2 constructs, Gly39* and Tyr746* that reflect alleles of NS patients, A5146 and B91, respectively, abrogate this interaction (Fig. 2a, b). The six mutations detected in *TNS2* did not abrogate interaction with MAGI2 (Supplementary Fig. 3B).

Second, MAGI2 also interacted with DLC1 (Fig. 2a, c). One mutant *MAGI2* construct Gly39* reflecting the mutation of NS patient A5146 abrogates this interaction (Fig. 2c), as confirmed in a confirmatory coIP (Supplementary Fig. 4).

Third, because TNS2 had been shown to interact with DLC1^19^, we performed coIP upon co-overexpression in HEK293T cells. We confirmed TNS2-DLC1 interaction (Fig. 2a), however, none of the *TNS2* or *DLC1* missense mutations identified in NS patients abrogated this interaction (Supplementary Fig. 5A, B).

Fourth, because we identified a *CDK20* mutation in patient A5013 with pTSNS, and because a related serine/threonine protein kinase, CDK5, was shown to interact with DLC1^22^, we tested whether CDK20 would likewise interact with DLC1. By coIP we show that in fact CDK20 interacts with DLC1 (Fig. 2a,d) which we confirmed in a coIP (Supplementary Fig. 6A). We found that the clone reflecting *DLC1* mutation Trp10* of individual A548 abrogated this interaction (Supplementary Fig. 6B). This interaction was confirmed by half endogenous Co-IPs in HEK293T cells (Supplementary Fig. 7A, B).

Finally, it was recently shown that the RhoGAP DLC1 interacts with caveolin-1 (CAV1) via DLC1’s START domain^23^ (Fig. 2a), and that *CAV1* expression is regulated by *EMP2*^24^ (Fig. 2a). This regulation was shown to be steroid sensitive^25^. Furthermore, we had shown that *EMP2* mutations were the only monogenic cause of SSNS known so far in humans^8^ as well as in a zebrafish model of SSNS^25^. To determine whether CAV1 could directly be involved in DLC1 regulation, we performed coIP between DLC1 and CAV1 by co-overexpression in HEK293T cell. We confirmed that DLC1 interacts with CAV1 (Fig. 2a, e), and showed that the mutant Lys1358Thr, reflecting the allele of NS patient A4967 and present in CAV-1 binding site in DLC1^23^, abrogated this interaction (Fig. 2e). To further test the effect of steroids on this interaction, we performed reciprocal coIP between co-overexpressed DLC1 and CAV1 in HEK293T cells that were pre- and post-treated with 100 uM of dexamethasone (Supplementary Fig. 8). However, this treatment did not alter the results shown in Fig. 2e. We demonstrated by co-overexpression in HEK293T cells that CAV1 also interacts with TNS2 and MAGI2 (Supplementary Fig. 9A, B).

In summary, we identified in patients with pTSNS recessive mutations in six different genes, the products of which mutually interact within a complex that contains the RhoGAP DLC1. The finding that specific mutations abrogated some of these interactions most likely reflects allele-specific pathogenic effects of these mutations.

Furthermore, we also investigated the endogenous expression of TNS2 and DLC1 by immunofluorescence microscopy (IF) in human podocytes and found that both TNS2 and DLC1 colocalize with an antibody labeling phosphotyrosine at focal and fibrillar adhesions, consistent with the role of focal adhesions in podocyte migration^26^ and the pathogenesis of NS^27,28^ (Supplementary Fig. 10). Additionally, by IF we show that upon overexpression, full length GFP-MAGI2 colocalizes with β-catenin at adherens junctions. In contrast, overexpression of GFP-MAGI2 Gly39* and Tyr746*, representing mutations in pTSNS, fail to localize at adherens junctions (Supplementary Fig. 11).

### Circular dichroism modeling of mutation in SAM domain of DLC1

Since one of the mutations (p.Ala456Val) in DLC1 is located in its SAM domain, we studied effect of this mutation on its secondary structure by performing circular dichroism (CD) assay. The wild type (WT) and mutant (Ala19Val in SAM domain) proteins did not show any difference in their CD spectrum indicating overall similar three dimensional fold (Supplementary Fig. 12A). We then recorded the melting spectrum of WT and mutant proteins and found that the denaturation temperature of WT SAM domain was near to 58 °C and the melting curve was sigmoidal indicating cooperative unfolding behavior. However, for Ala19Val SAM, there was loss of cooperativity and absence of sharp melting point (Supplementary Fig. 12B). Arg823Trp mutation in SAM domain of ANKS6 has been previously reported to cause structure destabilization and responsible for polycystic kidney disease^29^. Interestingly, both Arg823Trp (Arg64) mutation in ANKS6 and Ala456Val (Ala19) mutation in DLC1 are located in proximity in DLC1-SAM domain structure indicating a possible link of similar destabilization effect (Supplementary Fig. 12C, D).

### Knockdown of MAGI2 or CDK20 or DLC1 causes a decrease in cell migration rate

To elucidate the role of the genes of this RLSG regulatory module in the pathogenesis of pTSNS, we next studied cell migration rate, which is typically altered upon loss of function of NS-causing genes. We show that knockdown of *MAGI2* or *CDK20* in cultured human podocytes results in a reduced migratory phenotype (Supplementary Fig. 13A, B). Similarly, knockdown of *DLC1* in both IMCD3 cells and human podocytes also show a reduction in cell migration (Fig. 2f, Supplementary Fig. 14). This effect is found to be reversed when WT human DLC1 is transfected to IMCD3 cells (Fig. 2f). In contrast, four out of six *DLC1* mutants (Trp10*, Ala456Val, Ala1352Val, and Lys1358Thr), detected in pTSNS, failed to fully rescue this decreased migratory phenotype. The knockdown of *TNS2* did not show any effect on podocyte migration (Supplementary Fig. 15).

### RhoA regulation by this NS pathogenic module

Because defects in the regulation of Rho-like small GTPases have been implicated in the pathogenesis of monogenic SRNS^4,5,30^, we evaluated the effect of overexpression and transient knockdown of all six members of NS pathogenic module on the regulation of RhoA/Rac1/Cdc42 activity (Supplementary Fig. 16, Supplementary Table 5) and its potential impairment by the mutations detected in patients with pTSNS. We further evaluated the effect of stable knockdown of *TNS2* and *DLC1* in human podocytes, on the regulation of RhoA/Rac1/Cdc42 activity. Our results were consistent with the findings observed with the transient knockdown for these two genes (Supplementary Fig. 17).

No effect of overexpression or knockdown of *TNS2, CDK20, ITSN1,* or *ITSN2* was found on the regulation of active (GTP-bound) Rac1, with the exception of an increase of active Rac1 upon knockdown of *DLC1* (Supplementary Figs 16 and 17, Supplementary Table 5). This finding most likely indicates an effect of the known crosstalk between RhoA and Rac1 signaling.

**Fig. 3:**
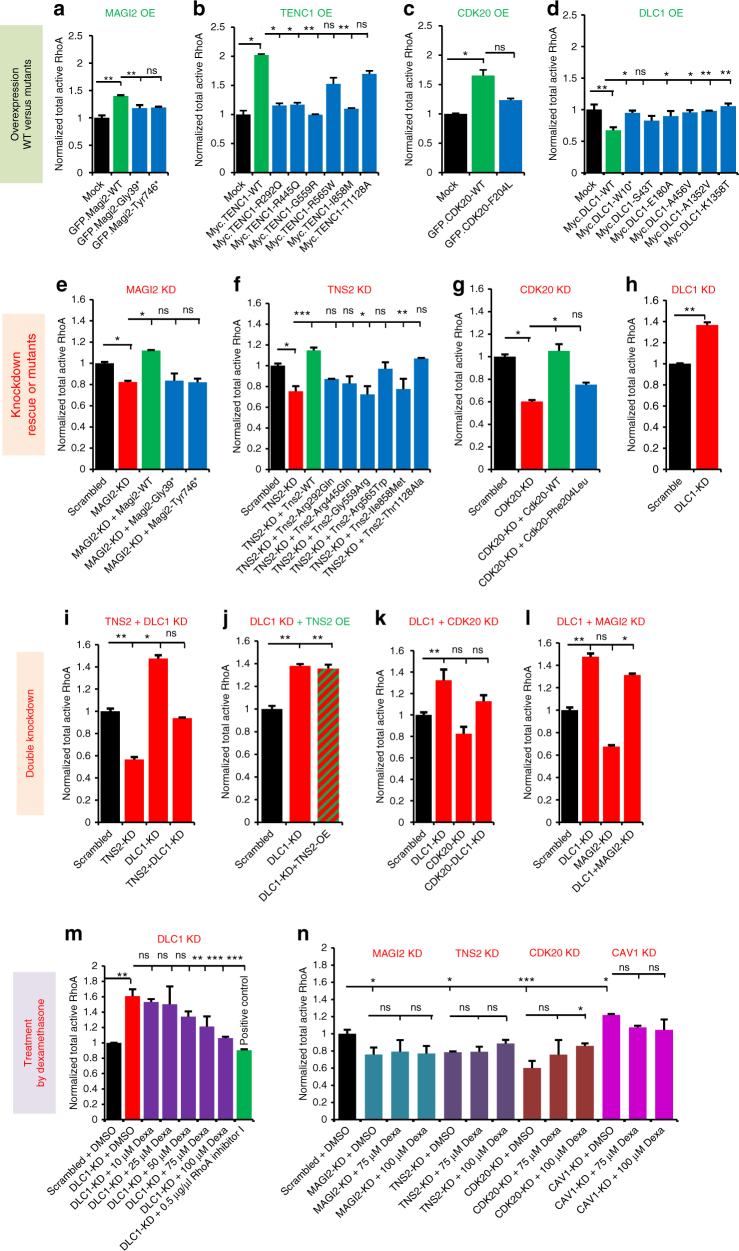
The novel RhoA regulatory network of MAGI2, TNS2, DLC1, CDK20, and CAV1 is a central part of the pTSNS pathogenesis. **a** Overexpression of GFP-tagged *MAGI2* wild type (WT) but not the two MAGI2 mutants from pTSNS patients resulted in a significant increase in active RhoA. Active RhoA was measured by G-LISA assay in HEK293T cells. **b** Myc-tagged *TNS2*-WT increased active RhoA levels upon overexpression in HEK293T cells as compared to Mock. However, all six *TNS2* mutant clones from pTSNS patients failed to increase active RhoA. **c** GFP-tagged *CDK20*-WT showed a significant increase in active RhoA level upon overexpression in HEK293T cells, while the mutant clone Phe240Leu, from a pTSNS patient, failed to do so. **d** Myc-tagged *DLC1*-WT showed a significant decrease in active RhoA level upon overexpression in HEK293T cells as compared to Mock. However, none of the six *DLC1* mutant clones from pTSNS patients showed this effect. **e** siRNA mediated knockdown of *MAGI2* decreased active RhoA. The overexpression of Magi2-WT rescued this effect while both the mutants from pTSNS patients did not. **f** siRNA mediated knockdown of *TNS2* decreased active RhoA. The overexpression of TNS2-WT rescued this effect, while four out of six TNS2 mutant clones from pTSNS patients did not. **g** siRNA mediated knockdown of *CDK20* decreased active RhoA. This effect was rescued by the overexpression of CDK20-WT, but not by the overexpression of mutant clone Phe240Leu. **h** siRNA mediated knockdown of *DLC1* showed a significant increase in active RhoA. **i** siRNA mediated knockdown of *TNS2* or *DLC1* in HEK293T cells decreased or increased active RhoA levels, respectively, as compared to scrambled control. The parallel knockdown of both *TNS2* and *DLC1* rescued this effect on active RhoA. **j** siRNA mediated knockdown of *DLC1* increased active RhoA and this effect was unaltered upon overexpression of *TNS2* in *DLC1* knockdown cells, suggesting that TNS2 modulates RhoA activity indirectly via DLC1. **k** siRNA mediated knockdown of *DLC1* or *CDK20* in HEK293T cells increased and decreased active RhoA levels, respectively, as compared to scrambled control. The combined knockdown of both *DLC1* and *CDK20* rescued this effect on active RhoA. **l** siRNA mediated knockdown of *DLC1* or *MAGI2* in HEK293T cells increased and decreased active RhoA levels, respectively, as compared to scrambled control. The combined knockdown of both *DLC1* and *MAGI2* failed to rescue this effect on active RhoA. **m** Treatment with dexamethasone (75 and 100 µM) inhibited in a dose-dependent manner the increase of active RhoA elicited by *DLC1* knockdown. **n** The change in active RhoA upon knockdown of *CDK20*, but not *MAGI2, TNS2*, or *CAV1* was found reversible by treatment of HEK293T cells with 100 µM dexamethasone. Error bars are defined as the standard error of at least three independent experiments. One-way ANOVA with Dunnet’s post hoc test was performed vs. control. Data are presented as the mean ± SEM. *P < 0.05, **P < 0.01, ***P < 0.001. ns, non significant

We found that overexpression of *MAGI2* increased active (GTP-bound) RhoA, whereas overexpression of the *MAGI2* mutants detected in patients with pTSNS lacked this effect (Fig. 3a, Supplementary Table 5). We tested the effect of TNS2 on RhoA regulation and found that TNS2 behaved similar to MAGI2 (Fig. 3b). Because we had shown that CDK20 interacts with the RhoGAP DLC1 (Fig. 2a, d), we tested the effect of CDK20 on RhoA regulation and found that CDK20 behaved similar to MAGI2 and TNS2 in that overexpression of CDK20 increased active RhoA, whereas overexpression of the *CDK20* mutant detected in patients with pTSNS lacked this effect (Fig. 3c). We then tested the effect on RhoA regulation of the RhoGAP DLC1, which interacts with MAGI2, TNS2, and CDK20. We found that DLC1 behaved opposite to MAGI2, TNS2, and CDK20 because overexpression of DLC1 decreased active RhoA. Overexpression of the *DLC1* mutants detected in patients with pTSNS again lacked this effect (Fig. 3d). Reciprocally, knockdown of *MAGI2, TNS2*,* and CDK20* decreased active RhoA, which was rescued by overexpression of respective WT cDNAs, whereas all or most of the mutants detected in patients with pTSNS lacked this effect (Fig. 3e–g, Supplementary Table 5). However, knockdown of *DLC1* was found to increase active RhoA (Fig. 3h). We thereby clearly showed that the mutations detected in individuals with pTSNS in the six genes of this newly discovered RhoA regulatory module are an important part of the pathogenesis of pTSNS. We also tested the effect of *CAV1* on RhoA activation and found that its overexpression decreases active RhoA, whereas its knockdown increased active RhoA (Supplementary Fig. 18).

We then tested the effect of double-knockdown of *TNS2* and *DLC1* on RhoA activity and found that the decrease in active RhoA by *TNS2* knockdown or increase in active RhoA by *DLC1* knockdown was normalized (Fig. 3i, Supplementary Table 5). To test if the TNS2 expression modulates the RhoA activity via DLC1, we knockdown DLC1, followed by overexpression of WT-TNS2 in HEK293T cells and found the increase was comparable to knockdown of DLC1 alone (Fig. 3j). This confirms that the TNS2 is upstream of DLC1 and works via DLC1 on RhoA regulation. Similarly, knockdown of DLC1 and CDK20 together (Fig. 3k) also showed that both CDK20 and MAGI2 affect RhoA regulation via DLC1, which plays a central role in this pathogenesis. In contrast, the double-knockdown of DLC1 and MAGI2 did not (Fig. 3l).

### Defects of RhoA regulation are mitigated by steroids

Since most of the individuals found here with mutations in *MAGI2, TNS2, DLC1, or CDK20* genes had clinically shown benefits by treatment with steroids, we next tested if steroid treatment with dexamethasone in different concentrations will show any the effect on the increase of RhoA activity upon *DLC1* knockdown in HEK293T cells. Wild type HEK293T cells showed no effect of dexamethasone treatment on active RhoA, while they responded to RhoA activators (red) and RhoA inhibitors (blue) as a positive controls (Supplementary Fig. 19). In contrast, treatment with dexamethasone at >75 µM abolished the effects on RhoA activation by knockdown of *DLC1* (Fig. 3m). We observed similar treatment effect of dexamethasone (>75 µM) on RhoA activation via knockdown of *CDK20* (Fig. 3n). However, no treatment effect of dexamethasone (<100 µM) was seen on RhoA activation via knockdown of *MAGI2, TNS2*, or *CAV1* (Fig. 3n). We further confirmed these findings in human podocyte cells (Supplementary Fig. 20). This indicates that the beneficial effect of steroids in pTSNS and mutations in these genes is likely mediated by the RhoA-regulating module.

### Discovery of ITSN1 and ITSN2 as two NS-relevant GEFs for Cdc42

**Fig. 4:**
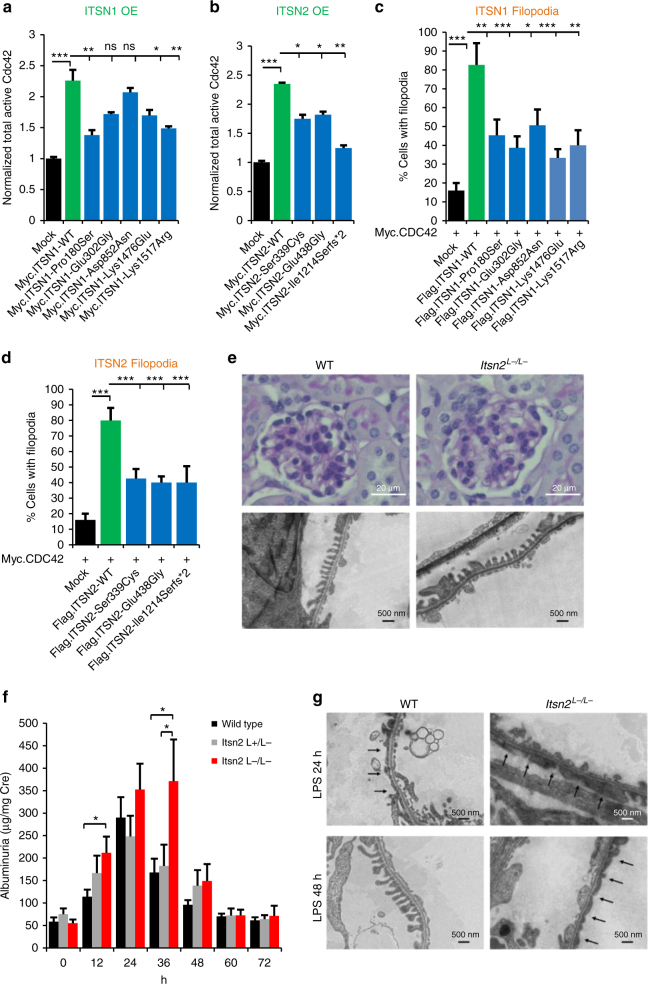
ITSN1 and ITSN2 are GEFs for Cdc42, regulating NS-related podocyte function, and *Itsn2*^−/−^ mice develop incompletely penetrant NS upon LPS injection. **a** Myc-tagged *ITSN1*-WT increased active Cdc42 levels upon overexpression in HEK293T cells as compared to mock, whereas three out of five *ITSN1* mutants from pTSNS patients failed to show any significant increase in active Cdc42 upon overexpression. **b** Active Cdc42 level was measured in COS7 cells. Myc-tagged *ITSN2*-WT overexpression increased active Cdc42 levels as compared to Mock. However, all three *ITSN2* mutants from pTSNS patients failed to show any significant increase in active Cdc42 upon overexpression. Error bars are defined as the standard error of at least four independent experiments. One-way ANOVA with Dunnet’s post hoc test vs. wild-type (WT) ITSN2 expressing control. **c** Filopodia induction in cultured human podocytes was quantified by counting the transfected cells with filopodia and showed as a percentage of cells. For each construct, 50 transfected cells from three independent experiments were analyzed. One-way ANOVA with Dunnet’s post hoc test vs. WT ITSN1 expressing control. Data are presented as the mean ± SEM. *P < 0.05, **P < 0.01, ***P < 0.001, ****P < 0.0001. **d** Filopodia induction in cultured human podocytes was quantified by counting the transfected cells with filopodia and showed as a percentage of cells. For each construct, 50 transfected cells from three independent experiments were analyzed. One-way ANOVA with Dunnet’s post hoc test vs. WT ITSN2 expressing control. Data are presented as the mean ± SEM. * *P* < 0.05, **P < 0.01, ***P < 0.001, ****P < 0.0001. **e** Kidneys of WT and *Itsn2* ^L−/L−^ mice were stained with periodic acid Schiff. The glomeruli of *Itsn2* ^*L−/L−*^ mice showed normal findings, similar to WT. **f** Urine albumin of *Itsn2* ^*L−/L−*^*, Itsn2* ^*+/L−*^, and WT mice was measured before and after LPS injection. Albuminuria level was increased in *Itsn2* ^*L−/L*−^ mice from 12 to 48 h after LPS injection compared with WT and *Itsn2* ^+/L−^ mice. Two-tailed Student’s *t*-tests. Data are presented as the mean ± SEM. *P < 0.05. **g** Representative electron microscopy images. LPS injection-induced foot process (FP) effacement in both WT and *Itsn2* ^*L−/L−*^ mice within 24 h. At 48 h after LPS injection, FP effacement was still observed in *Itsn2* ^*L−/L−*^ mice, although WT mice recovered from FP effacement

We here discover recessive mutations in *ITSN1* and *ITSN2* as causing NS. Both proteins are members of the guanine exchange factor (GEF) family of proteins that activate Cdc42^31^ (Fig. 2a). To test whether loss of function of ITSN1 and ITSN2 GEF activity causes a nephrosis phenotype in humans, we examined the effect of mutations identified in patients with pTSNS on the active states of Cdc42. We show that overexpression of WT-*ITSN1* results in a significant increase in active Cdc42 in HEK293T cells (Fig. 4a) or human podocytes (Supplementary Fig. 21). All of the *ITSN1* mutants detected in patients with pTSNS lacked this effect, demonstrating its relevance for the pathogenesis of NS. Similar results were obtained for *ITSN2* where all the mutations detected in pTSNS patients lacked the effect of Cdc42 activation (Fig. 4b). Filopodia, thin actin-rich plasma-membrane protrusions, play an important role in cell migration and wound healing^32^ and Cdc42 is a key protein involved in filopodia formation^33^. Thus, we examined the effect of *ITSN1* and *ITSN2* mutations on filopodia induction in cultured human podocytes. Human podocyte cells were co-transfected with Myc-Cdc42 and FLAG tagged WT-*ITSN1*, five mutants discovered in pTSNS patients (Pro180Ser, Glu302Gly, Asp852Asn, Lys1476Glu, and Lys1517Arg) or flag-mock and images were captured (Supplementary Fig. 22). WT-*ITSN1* transfected cells induced dense filopodia all around the cell surfaces (Supplementary Fig. 22). Overexpression of *ITSN1* mutants resulted in a significant decrease in the ratio of cells with filopodia compared to WT-*ITSN1* (Fig. 4c). Similar loss of filopodia induction was observed for overexpression of *ITSN2* wild type vs. three mutants discovered in pTSNS patients (Ser339Cys, Glu438Gly and Ile1214Serfs*2) (Fig. 4d, Supplementary Fig. 23). We thus demonstrated that the *ITSN1* and *ITSN2* mutations detected from the pTSNS families cause loss of function in Cdc42-GEF activity and also inhibit filopodia induction in cultured human podocytes cells. The other genes were not tested because their overexpression or knockdown altered RhoA activity, which induces focal adhesions, rather than Cdc42, which induces filopodia.

### Generation of Itsn2 deficient mice

To evaluate the pathogenic roles of *ITSN2* mutations in the pTSNS disease phenotype, we generated *Itsn*-*L* KO mice. In the KO mice, a region including exon 31 of *Itsn2* (NC_000078) was replaced with the targeting vector throughout the entire body (Supplementary Fig. 24A). This region encodes a part of the DH domain, affecting Cdc42-GEF activity. The expression level of Itsn2-S and Itsn2-L mRNA in various organs were determined by reverse transcription PCR (RT-PCR; Supplementary Fig. 24B). Itsn2-S and Itsn2-L mRNA were expressed ubiquitously in wild type mice. In the homozygous KO (*Itsn2*^L−/L−^) mice, Itsn2-L mRNA was not detected (Supplementary Fig. 24B). *Itsn2* ^L−/L−^ mice were normal in appearance, growth, development, and fertility. There was no difference between *Itsn2* ^L−/L−^ and wild-type mice in the histologic findings of the kidneys (Fig. 4e) and in the level of urinary protein (Fig. 4f at 0 h) during the course of natural history. These results suggested that Itsn2-L is not necessary for kidney development in mice. Patients with NS often relapse after an upper respiratory tract infection^34^. We hypothesized that some interventions that mimic infectious events may trigger the onset of a nephrosis phenotype in *Itsn2*^L−/L−^ mice. We used the LPS model of transient nephrotic syndrome, in which foot process effacement and massive proteinuria of wild-type mice develop within 12–24 h and return to baseline levels within 72 h^35^. The time course of albuminuria was measured in wild type, *Itsn2* ^+/L−^ and *Itsn2* ^L−/L−^ mice at various time points before and after the injection of LPS (Fig. 4f). There was no difference in urine albumin between wild-type and *Itsn2*^L−/L−^ mice before the injection of LPS. Urine albumin was increased in *Itsn2*^L−/L−^ mice from 12 to 48 h after LPS injection, with statistically significant differences at 12 and 36 h compared with WT mice and *Itsn2* ^+/L− ^mice. At 36 h of observation, albuminuria continued to be elevated in *Itsn2* ^L−/L−^ mice, while it started to decline in WT and *Itsn2* ^+/L−^ mice. In all groups, urine albumin returned to baseline at 60 h. To confirm whether the urinary phenotype reflected pathologic findings, we examined the electron microscopy of mice kidneys. LPS injection induced foot process (FP) effacement in both WT and *Itsn2*^L−/L−^ mice within 24 h. At 48 h after LPS injection, FP were almost recovered in WT mice, while diffuse FP effacement was observed in *Itsn2*^L−/L−^ mice (Fig. 4g). We thereby demonstrated that ITSN2-L knockout caused delayed recovery from podocyte injury. This finding adds yet another example of a pathogenic role of small Rho-like GTPases (here Cdc42), in which there is incomplete penetrance of the disease phenotype, analogous to the partially treatment sensitive phenotype (pTSNS) seen in mutations in the RhoA regulatory module members—*MAGI2, TNS2, DLC1*, and *CDK20* (Fig. 2a).

## Discussion

We here discover recessive mutations of *MAGI2, TNS2, DLC1, CDK20, ITSN1*, and *ITSN2* genes as novel causes of NS. Whereas over 50 monogenic genes have been published to cause steroid resistant nephrotic syndrome (SRNS), if mutated, there is virtually no monogenic cause known for steroid sensitive nephrotic (SSNS) syndrome, the latter including steroid dependent (SDNS) and frequently relapsing nephrotic syndrome (FRNS). Thus, SRNS and SSNS have always appeared as molecularly and functionally completely separate entities. Therefore our finding that nine of the 12 patients with mutations in one of the six genes of the “Rho-like small GTPase regulatory cluster” had a partially treatment responsive form of nephrotic syndrome is quite striking and has not been described in a similar way before. This finding confirms the unique role of the pathway discovered that maybe amenable to therapy.

RhoA/Rac1/Cdc42 regulation has been implicated in SRNS before^36^ and few genes that code the regulators of Rho GTPases were found to be associated with human glomerular disease. We showed that *ARHGDIA*, which encodes a GDI for Cdc42 and Rac1, causes autosomal recessive SRNS^4^. In addition, patients with NS and mutations in *KANK2*, which encodes kidney ankyrin repeat-containing protein 2, exhibited increased active RhoA in podocytes^5^. Furthermore, we demonstrated that mutations in *FAT1* cause familial focal segmental sclerosis (FSGS) via defective Rho GTPases^30^. Podocyte-specific loss of Cdc42 in mice has been described to lead to foot process effacement and congenital proteinuria^37^. It has been suggested that there is strong crosstalk between the three Rho-like small GTPases RhoA, Rac1, and Cdc42^38^. Therefore, we here evaluated activation of all three Rho-like small GTPases by G-LISA for each of the six novel monogenic causes of pTSNS. In contrast to previous findings showing a role of Rac1 in SRNS of humans, mice and zebrafish^4^, and a role of Cdc42 in NS in mouse models^37^, we here discovered that for four different novel genes (*MAGI2, TNS2, CDK20*, and *DLC1*), which if mutated cause pTSNS, modulation of activated RhoA rather than Rac1 or Cdc42 is central to the pathogenesis (Supplementary Table 6). This may indicate that in pTSNS caused by mutations in these four genes impairment of the RhoA-driven function of focal adhesions is more relevant for the pathogenesis than impairment of Rac1-driven lamellipodia formation.

Mechanisms of steroid action in NS have been a conundrum for the six decades of their use. Interestingly, we found that the effects of KD of *DLC1* or *CDK20* on RhoA activation in human podocytes and HEK293T cells were abolished by steroid treatment with dexamethasone. We here generate evidence of steroid action on podocyte function indicating that this newly delineated RLSG regulatory module mediates the beneficial effect of steroids in patients with pTSNS and mutations in these genes. We cannot evidently answer why steroids help cure NS in some patients, and it has been only known that steroids may influence the transcription of a large number of genes by virtue of their interaction with intracellular receptors. However, our data suggests that steroids might target a specific protein or modulator of protein, which is most likely a part of the complex described in this manuscript, rather than the transcriptional target through the receptors. Further studies of this protein interaction module may reveal the specific target(s) of steroid action in podocytes.

*Tns2*-deficient mice have been previously described to develop mild glomerular injury on a DBA/2 background, but not on a C57BL/6J or a 129/SvJ background, suggesting that glomerular injury by the deletion of *Tns2* was modified by the genetic background^39^. In mice, LPS stimulates toll-like receptor-4 (TLR-4) and upregulates CD80 expression on podocytes, resulting in foot process effacement and transient proteinuria^35^. Here, we generated the *Itsn2-L* knockout mice with partially penetrant proteinuria following LPS injection. This mouse model exhibits an NS phenotype that is at an interphase between plain steroid resistance and steroid sensitivity. We observed that the individuals with *ITSN2* mutations had frequently relapsing NS while the *Itsn2-L* knockout mouse showed inducible and transient proteinuria. In humans, the severity of SSNS varies, where some patients show complete remission with steroid therapy while some have frequently relapsing NS. The study by Wharram et al. published in *J. Am. Soc. Nephrol.* in 2005^40^ described that the different levels of podocyte depletion assess the level of glomerulosclerosis in the transgenic rat model. This means that inducible, partially sensitive, and frequently relapsing NS are all on a spectrum with steroid-resistant NS. Furthermore, SSNS maybe a multifactorial disease and not a simple Mendelian disorder. It may also be influenced by multiple modifying genes and environmental factors such as infections and nature of host immune responses, besides mutation in a particular gene. Therefore, the nephrotic phenotype of *Itsn2-L* knockout mice may vary greatly depending on genetic background of mouse strain and environmental factors, configuring a phenotypic spectrum of the *Itsn2*-related NS. Therefore, it is not surprising that *Itsn2-L* knockout mouse did not mimic the symptoms of the patients with *ITSN2* mutations.

In summary, we detected recessive mutations in cohort of individuals with partially treatment sensitive NS, which places this pathogenesis at the interphase between SSNS and SRNS. Definition of this pathogenic pathway opens inroads into defining therapeutic targets for SRNS, for which currently no efficient treatment exists.

## Methods

### Study participants

We obtained blood samples and pedigrees following informed consent from individuals with NS. Approval for human subjects’ research was obtained from Institutional Review Boards of the University of Michigan, the Boston Children’s Hospital and other local IRBs. The diagnosis of NS was based on published clinical criteria. This study was also approved by the Ethical Committee of Tohoku University Graduate School of Medicine. Written informed consent was obtained from all members of the Japanese families and the individuals with SSNS for the use of their clinical data and blood samples.

### Linkage analysis

For genome-wide homozygosity mapping the GeneChip® Human Mapping 250 k *Sty*I Array from Affymetrix was used. Non-parametric LOD scores were calculated using a modified version of the program GENEHUNTER 2.1^41,42^ through stepwise use of a sliding window with sets of 110 SNPs and the program ALLEGRO^43^ in order to identify regions of homozygosity as described^10,44^ using a disease allele frequency of 0.0001 and Caucasian marker allele frequencies.

### Whole exome sequencing

Whole exome sequencing (WES) and variant burden analysis was performed as described^45^. In brief, genomic DNA was isolated from blood lymphocytes and subjected to exome capture using Agilent SureSelect™ human exome capture arrays (Life technologies^TM^) followed by next generation sequencing on the Illumina^TM^ sequencing platform. For family from Japan, exon capture was performed with SureSelectXT Human All Exon V4 (51 Mb) Kits (Agilent Technologies). Exome libraries were sequenced on a HiSeq2000 (Illumina) according to the manufacturer’s instructions. Paired reads were aligned to the hg19 human reference using Novoalign V2.08.05 (http://novocraft.com), and single nucleotide variants (SNVs) and insertions and/or deletions (indels) were called using the Genome Analysis Toolkit (GATK) v1.6-13^46^.

### Targeted sequencing

DNA was obtained from 153 individuals with SSNS by the group from Japan. The 153 patients screened for *ITSN2* mutations were screened by panel sequencing rather than by whole exome sequencing. We designed a custom panel of 21 genes (total target bases: 183.44 kbp, Supplementary Table 7) using the SureDesign Tool (Agilent Technologies). Target capture was performed with the HaloPlex target enrichment system (Agilent Technologies). Libraries were sequenced on a MiSeq or HiSeq2000 (Illumina). Mapping and variant call were performed using SureCall v2.1.0.21. Variants in *ITSN2* were manually verified using an Integrative Genomics Viewer (IGV), and nonsynonymous mutations were retained and filtered for a MAF < 0.01 in the 1000 Genomes database and HGVD.

### Mutation calling

Sequence reads were mapped against the human reference genome (NCBI build 37/hg19) using CLC Genomics Workbench (version 6.5.1) software (CLC bio). Variants with minor allele frequencies <1% in the dbSNP (Version 137) database were selected and annotated for impact on the encoded protein and for conservation of the reference base and amino acid among orthologs across phylogeny. Mutation calling was performed by geneticists/cell biologists, who had knowledge of the clinical phenotypes and pedigree structure, as well as experience with homozygosity mapping and exome evaluation.

### High-throughput mutation analysis by array-based multiplex PCR and NGS

We used PCR-based 48.48 Access Array microfluidic technology (Fluidigm^TM^) with consecutive next generation sequencing. We applied a 12-fold primer multiplexing approach allowing PCR-based amplification for 48 DNA samples simultaneously in 576 amplicons^12,13^. A total of 2000 individuals with NS were analyzed, among them subset of 800 individuals with SSNS. After amplification of all targeted coding and splice site regions, sample-derived products were indexed with 384 different 10 bp-barcodes in a subsequent PCR. Finally, 2 × 250 bp paired-end sequencing was performed on an Illumina^TM^ MiSeq instrument. Bioinformatic analysis was conducted using CLC-Genomics-Workbench^TM^ software. Potential mutations were confirmed by Sanger sequencing and evaluated for segregation.

### cDNA cloning

Rat *Magi2* (*S-SCAM*) full-length cDNA was gifted by Dr. Min Loo, Yonsei University College of Medicine, Korea. Human *TNS2* isoform 2 full-length cDNA was subcloned by PCR from human full-length cDNA (cDNA clone MGC: 165058, IMAGE: 40148847). Mouse *TNS2* was subcloned from mouse *TNS2* full-length ORF cDNA (Cat No: MR211954, Origene). Human *DLC1* subcloned from human fetal brain cDNA library (Clontech). Human *CDK20* full-length was subcloned from human full-length cDNA (cDNA clone MGC: 3757, IMAGE: 3605918). Mouse *Cdk20* was subcloned from mouse full-length Cdk20 (cDNA clone, MGC: 38901, IMAGE: 5362198). Human *ITSN1* full-length was subcloned from human full-length cDNA (cDNA clone MGC: 134949 IMAGE: 40073781). Expression vectors were produced using LR clonase (Invitrogen®) following the manufacturer’s instruction. The coding sequences of human *ITSN2-L* was purchased from Origene, and the *ITSN2-L* with the N-terminal FLAG tag (FLAG-ITSN2-L) or FLAG (mock) was subcloned into pCAGEN using the In-Fusion® HD Cloning Kit (TaKaRa, Clontech). The following expression vectors were used in this publication: pRK5-N-Myc, pCDNA6.2-N-GFP, pCMV6-AN-DDK (Origene) or pCAGEN. pCAGEN was a gift from Connie Cepko (Addgene plasmid # 11160)^47^. Clones reflecting the mutations identified in individuals with NS were introduced in the cDNA constructed by using the Quick change II XL site-directed mutagenesis kit, Agilent Technologies or by the PrimeSTAR® Mutagenesis Basal Kit (TaKaRa). Final sequences were confirmed by sequencing. pRK5-Myc-Cdc42-WT was a gift from Gary Bokoch (Addgene plasmid # 12972).

### Cell lines

Experiments shown in this publication were performed in HEK293T cells, and immortalized human podocytes. HEK293T cells were purchased from the ATCC biological resource center. Human immortalized podocytes were a kind gift from Moin Saleem, University of Bristol, Bristol, UK, and were cultured as previously described^48^. Cell lines were tested for mycoplasma contamination on a quarterly basis.

### Coimmunoprecipitation

Coimmunoprecipitation experiments were performed as described previously^49^. Briefly, cell lysates were pre-cleared with protein G or A beads. Then, cell lysates were mixed with the appropriate antibodies and incubated overnight at 4 °C in lysis buffer containing the complete protease inhibitor mixture. Immune complexes were collected by binding to mixed protein G or A beads and washed four times with lysis buffer prior to immunoblotting. Coimmunoprecipitation of GFP fusion proteins was performed using Chromotek-GFP-Trap® Agarose Beads, allele bioscience.

### Knockdown in HEK293T cells and human podocytes

Knockdown efficiencies for respective siRNAs in HEK293T cells for *TNS2, DLC1, CDK20*, and *ITSN1* are shown in Supplementary Fig. 25 and for *CAV1* in Supplementary Fig. 26. Efficiency of transient knockdown of *MAGI2*, *TNS2*, *DLC1*, *CDK20,* and *ITSN1* in undifferentiated human podocytes was confirmed by measuring mRNA levels by real-time PCR using TaqMan probes (Supplementary Fig. 27). All target sequences are listed in Supplementary Table 8. Knockdown experiments were performed 48 h after transfection. shRNA targeting human *TNS2* and *DLC1* were subcloned into pSirenRetroQ for retroviral transduction using HEK293T cells. Forty eight hour after transduction, puromycin was added to the medium at a final concentration of 4 μg/ml for selection of transduced cells. See Supplementary Table 8 for target sequences and Supplementary Fig. 28 for knockdown efficiency.

### Cell migration assay

Briefly, the migration assays were performed using IncuCyte™ video-microscopy system (Essen Biosciences) according to the manufacturer’s instructions. IMCD3 cells or human podocytes were transfected with siRNAs for *DLC1, MAGI2, CDK20*, or *TNS2*. For rescue experiments (Fig. 2f), knockdown of Dlc1 in IMCD3 cells was followed by transfection with mock-myc or human *DLC1* WT or mutants. Results were plotted as percentage of wound confluence (relative podocyte migration) vs. time. Each experiment was performed at least in triplicates, and repeated two times independently. Results are presented as mean with standard deviation.

### G-LISA Rho, Rac, and Cdc42 activation assays

Cells were transfected in six-well plates with WT constructs or mock using Lipofectamine-2000 for overexpression experiments, or with siRNA against the target or scrambled control using Lipofectamine-RNAiMAX for knockdown experiments. Transfected cells were incubated in DMEM (HEK293T) or RPMI (human podocytes) with 10% fetal bovine serum for 8 h and then in serum-free medium for 24 h. Rho, Rac, or Cdc42 activity was determined using a colorimetric G-LISA Rho or Rac or Cdc42 Activation Assay Biochem kit (Cytoskeleton), according to the manufacturer’s instructions.

### Measurement of filopodia induction in human podocytes

Human podocyte cells on glass coverslips were transfected with CDC42 constructs and *ITSN1* or *ITSN2* constructs or pCAGEN-FLAG-mock using Lipofectamine 2000® in six-well plates and were incubated in RPMI with 10% fetal bovine serum for 8 h and then in serum-free medium for 24 h. Cells were fixed in 4% paraformaldehyde (PFA) for 10 min and containing 0.25% Triton X-100. Cells were stained for 60 min at room temperature (RT) with mouse monoclonal anti-FLAG antibodies (1:500, Sigma-Aldrich, F3165) and rabbit polyclonal anti-Myc antibodies (1:500, santa cruz, sc-789), washed with phosphate-buffered saline (PBS) and incubated for 1 h at RT with Alexa Fluor 647 anti-mouse secondary antibody (1:500, life technologies, A31571), Alexa Fluor 594 anti-rabbit secondary antibody (1:500, life technologies, A21207) and Alexa Fluor 488 Phalloidin (1:40, Invitrogen, A12379). Coverslips were then mounted on slides with ProLong Gold antifade reagent with DAPI (Invitrogen). Confocal imaging was performed using Leica SP5X system with an upright DM6000 microscope and images were processed with the Leica AF software suite. Filopodia were defined as thin, finger-like protrusive structures^32^. The ratio of cells with filopodia was calculated.

### Circular dichroism (CD)

The WT-DLC1 SAM domain (amino acid number 17–76) was cloned in pETM vector as described^50^. A19V point mutation was incorporated in WT construct by site directed mutagenesis. The mutated DNA was transformed into *Escherichia coli* XL1-blue cells and plated on antibiotic containing agar plate. The correct in frame mutations were confirmed by sequencing of selected clones. The pET-M plasmids were transformed into *E. coli* BL21 (DE3) cells and the cells were grown in LB broth at 37 °C. Protein expression was induced by adding 1 mM IPTG. The cells were harvested and lysed by sonication in lysis buffer containing 20 mM Tris-HCl (pH 8), 200 mM NaCl, 5 mM DTT, 1% Triton X-100, 5% Glycerol, and protease inhibitor (Roche). The cleared lysates was transferred to previously washed Ni-NTA beads for overnight binding. Protein with 6x His tag was eluted from beads in two step elution in buffer 20 mM Tris-HCl (pH 8), 150 mM NaCl, 5% Glycerol, 5 mM DTT, and 200–300 µM Immidazole. The eluted protein was loaded on pre-equilibrated Superdex 75 column for further purification by size exclusion chromatography. CD spectrum was collected for WT and mutant protein samples on a Jasco J-810 CD spectropolarimeter with protein concentration ~30 µM in 10 mM Tris (pH 8) and 75 mM NaCl. Spectra were recorded in a cuvette of path length 0.1 mm. The melting curves were obtained by recording the CD signals at 222 nm across the temperature range from 20 to 90 °C. The reading was taken at every 2 °C increase of temperature.

### Antibodies

For immunofluorescence experiments the following primary antibodies were used: rabbit anti-TNS2 (Sigma, HPA034659, 1:100); mouse anti-DLC1 (BD Biosciences, clone 3, 1:200); rabbit anti-ITSN1 (Abcam, ab118262, 1;100); and rabbit anti-ITSN2 (Abcam, ab176592, 1:100). Donkey anti-goat Alexa 488 (1:500) and Alexa-594 (1:500) conjugated secondary antibodies, and DAPI (4ʹ,6-Diamidino-2-Phenylindole, Dihydrochloride, 1:10,000) were obtained from Invitrogen (Supplementary Fig. 29). For immunoblotting the following primary antibodies were used: CDK20 rabbit polyclonal (Novus, NBP1-91214, 1;1000); ITSN1, rabbit polyclonal (Abcam, ab118262, 1:1000). Antibodies against *TNS2* or *DLC1* used here have been described^51,52^ (Supplementary Fig. 25). HRP labeled secondary antibodies were purchased from Santa Cruz.

### Immunofluorescence and confocal microscopy in cell lines

For immuno-staining human immortalized podocytes were seeded on coverglasses, and grown at permissive temperature. For overexpression studies human podocytes were transiently transfected using lipofectamine 2000® following the manufacturer’s instructions. Experiments were performed 24–48 h after transfection. Cells were fixed and permeabilized for 10 min using 4% paraformaldehyde and 0.25%Triton-X100. After blocking, cells were incubated over-night at 4 °C. The cells were incubated in secondary antibodies for 90 min at room-temperature, followed by 5 min staining with 1 × DAPI/PBS. Confocal imaging was performed using Leica SP5X system with an upright DM6000 microscope and images were processed with the Leica AF software suite.

### *Itsn2*-L knockout mice

All animal experiment protocols were approved by The Institutional Animal Care and Use Committee of Tohoku University. Mice were housed in a temperature- and humidity-controlled room, with a 12-h light/dark cycle and were allowed access to food and water ad libitum. Generation of the *Itsn2*-L knockout mice were entrusted to Unitech (Chiba, Japan). The targeting vector replaced a region that included exon 31 of *Itsn2* (NC_000078; Supplementary Fig. 24) so that the *ITSN2* long isoform, which has Cdc42-GEF activity, was absent throughout the entire body of the mice. The knockout mice were produced on a background of C57BL/6. Genotyping was performed by real-time PCR using a HybProbe assay as previously described^53^. The primers and probes were designed as follows: 5ʹ-CTTCCTCACTGAAGCAGAC-3ʹ (forward primer for wild-type), 5ʹ-TGCTCCAGACTGCCTTG-3ʹ (forward primer for knockout), 5ʹ-TGCTCCTGCTTGGAGAT-3ʹ (reverse primer), 5ʹ-CGGGGGCTGCTCAGCAA-FITC-3ʹ (upstream probe for wild-type), 5ʹ-TCAAGCTTATCGATGATATCA-FITC-3ʹ (upstream probe for knockout), 5ʹ-LCRed640-ACAACTTCCAACTCTGTTCTGTC-P-3ʹ (downstream probe). Template DNA was extracted from tails. To confirm the loss of *Itsn2*-L mRNA transcripts, RT-PCR was performed. Total RNA was isolated from the kidney, liver, heart, and brain of Itsn2-knockout mice and wild type mice using an RNeasy Mini Kit (Qiagen). Using a PrimeScript II High Fidelity RT-PCR Kit (TaKaRa Bio), 500 ng of RNA was reverse transcribed. Total RNA was amplified using the following primer set: 5ʹ-CACGCTGTATGTCAAGTGATTGC-3ʹ (forward primer) 5ʹ-CATTGGGTCACTGTTGACTTGG-3ʹ (reverse primer for Itsn2 short) 5’-CTTCAGTGAGGAAGCCTGACTCAG-3’ (reverse primer for *Itsn2* long).

### LPS-induced proteinuria and electron microscopy

Transient proteinuria was induced by LPS injection as described^54^. We injected 4- to 6-week-old female C57BL/6 (*n*=18), Itsn2^+/L−^ (*n*=10), and Itsn2^L−/L−^ (*n*=10) mice with LPS (200 µg intraperitoneally (i.p.); SIGMA Aldrich). Urine samples were obtained from individual mice at various time points before (0 h) and after (12, 24, 36, 48, 60, and 72 h) the injection of LPS. Urinary albumin and creatinine were measured using an Albuwell M kit and the creatinine companion kit (Exocell) according to the manufacturer’s protocol. Albuminuria was calculated as micrograms of albumin per milligram creatinine. Kidneys were dissected from WT and *Itsn2*-L^−/−^ mice 24 and 48 h after LPS injection. Specimens for EM were fixed with 2% PFA and 2.5% glutaraldehyde, washed with 0.1 M cacodylate buffer, and incubated with 1% OsO_4_ for 90 min. The sections were counterstained with 1% uranyl acetate for 50 min, dehydrated in a graded series of ethanol (50, 60, 70, 80, 90, and 95%), and finally embedded in Epon 812. Ultrathin sections (80 nm) were cut with a Leica EM UC7 (Leica) and mounted in copper grids. Images were obtained using a transmission electron microscope (H-7600; Hitachi High-Technologies). The mice experiments were not blinded.

### Statistical analysis

Statistical analysis was performed with Graph Pad Prism. One-way ANOVA with Dunnet’s post hoc test was performed. Displayed in the figure are the mean values of all technical replicates for each of the independent experiments. Error bars represent s.d. *P* values <0.05 were considered statistically significant.

### Bioinformatics

Genetic location is according to the February 2009 Human Genome Browser data (http://www.genome.ucsc.edu).

### Data availability

All data generated or analyzed during this study are available in this published article (and its Supplementary Information Files) or from the corresponding author upon reasonable request.

## Electronic supplementary material

Supplementary Information
