# Supplementary material for: Mutations in six nephrosis genes delineate a pathogenic pathway amenable to treatment
Source: Nat Commun. 2018 May 17;9:1960. doi: 10.1038/s41467-018-04193-w (PMC5958119; doi:10.1038/s41467-018-04193-w)
Supplement: Supplementary file 1 — Supplementary Information [file 41467_2018_4193_MOESM1_ESM.pdf]

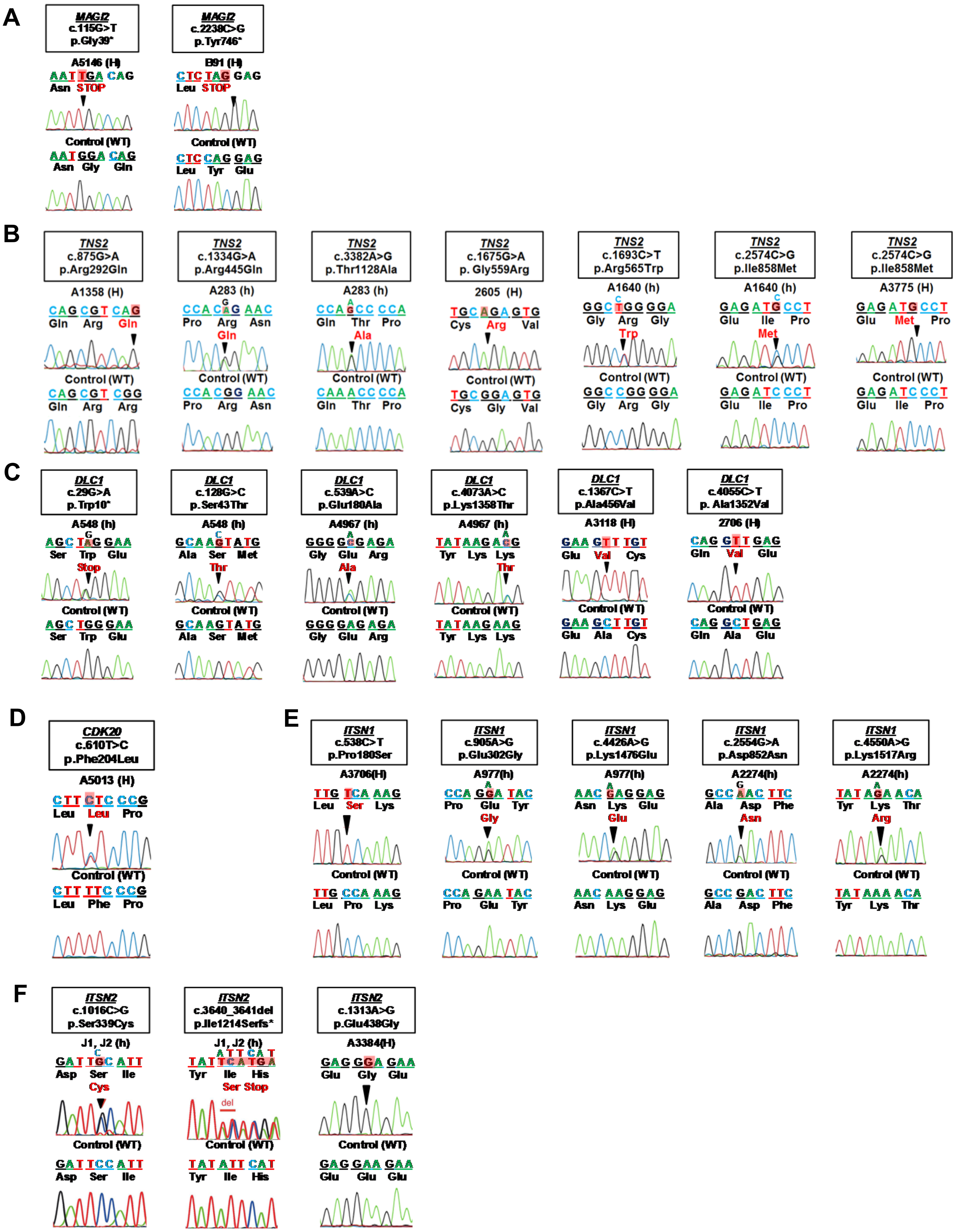

Supplementary Figure 1. Sequencing traces of individuals with mutations in *MAGI2* (A), *TNS2* (B), *DLC1* (C), *CDK20* (D), *ITSN1* (E) and *ITSN2* (F) .

Arrowheads denote altered nucleotides. H, homozygous mutation; h, heterozygous mutation; WT, wildtype.

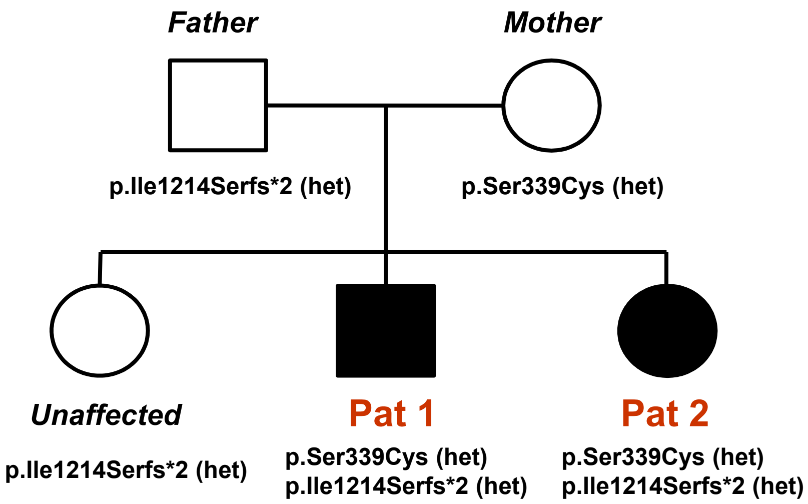

Supplementary Figure 2. Pedigree of Japanese family (Pat 1 and Pat 2) with pTSNS identified with mutations in *ITSN2*.

Parents and the older sister are healthy and showed normal urinary findings.

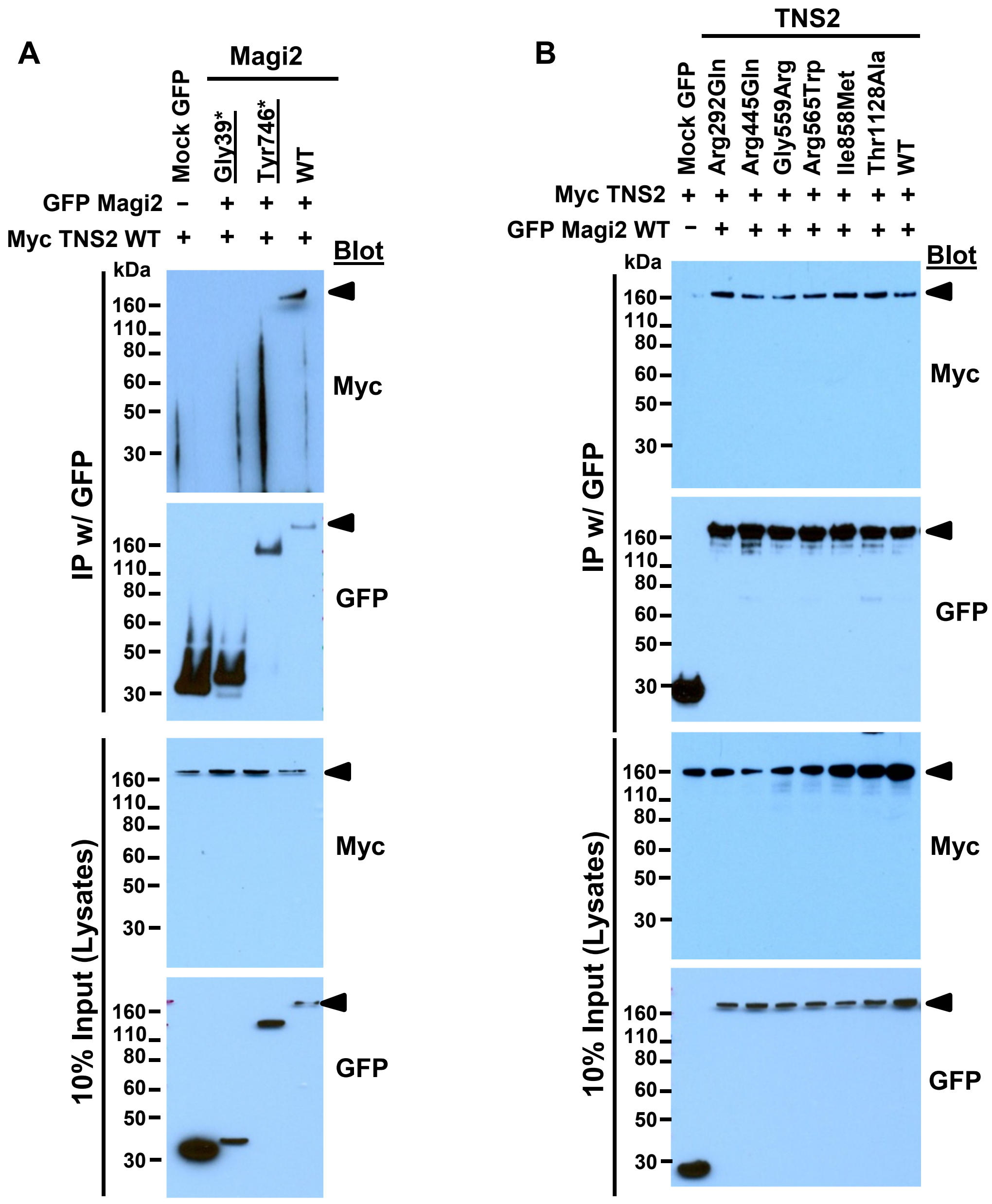

Supplementary Figure 3. TNS2 interacts with Magi2 upon co-overexpression in HEK293T cells.

(A) Both mutant rat *Magi2* clones, Gly39* and Tyr746* (underlined), reflecting alleles identified in individuals with NS abrogate this interaction. This is the confirmatory experiment to **Fig. 2b**.

(B) Mutant clones reflecting alleles of human *TNS2* identified in individuals with NS do not abrogate the interaction with rat *Magi2*. WT, wild type.

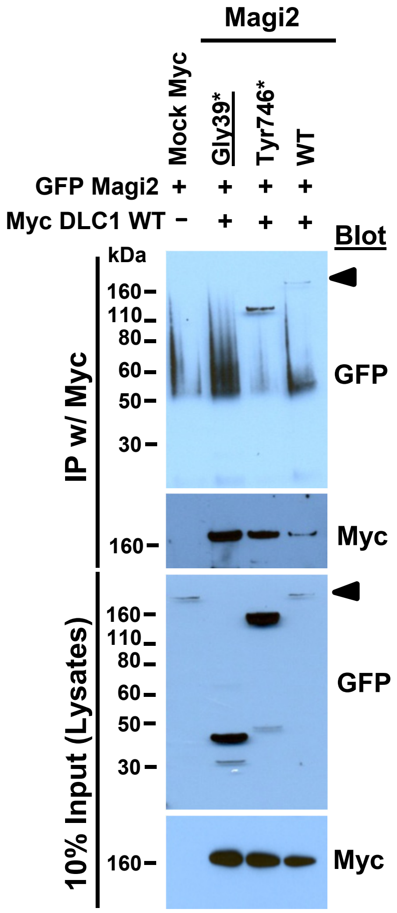

Supplementary Figure 4. DLC1 interacts with Magi2 upon co-overexpression in HEK293T cells.

One mutant clone Gly39* (underlined), reflecting an allele of *MAGI2* identified in individual with NS abrogates this interaction. This is the confirmatory experiment to **Fig. 2c**.

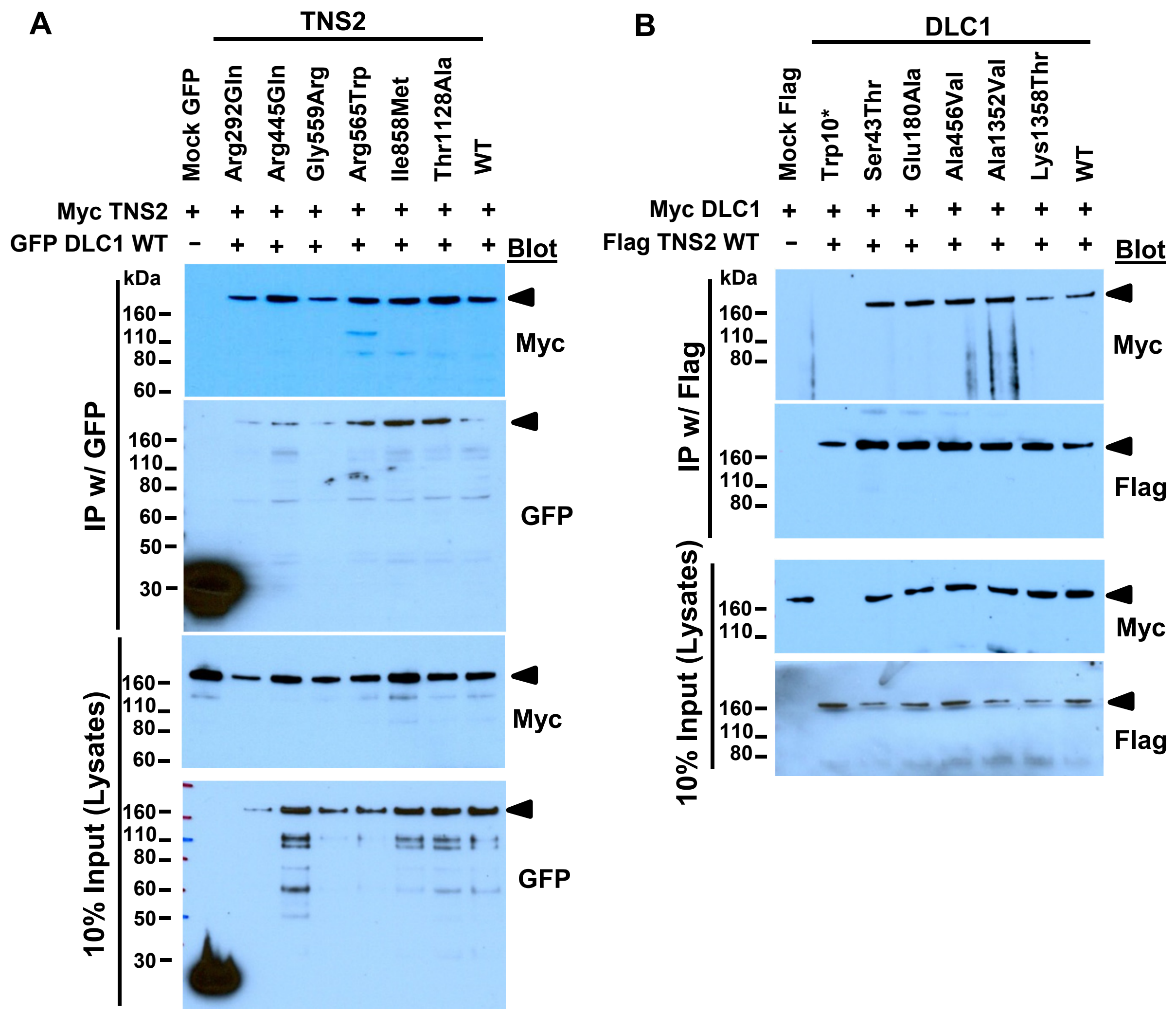

Supplementary Figure 5. DLC1 interacts with TNS2 upon co-overexpression in HEK293T cells.

(A) Myc tagged TNS2 interacts with GFP tagged DLC1 upon co-overexpression in HEK293T cells. None of the mutant clones reflecting alleles of *TNS2* identified in individuals with NS abrogate this interaction.

(B) Myc tagged DLC1 interacts with Flag tagged TNS2 upon co-overexpression in HEK293T cells. None of the clones reflecting alleles of *DLC1* identified in individuals with NS, except Arg10*, abrogate interaction with TNS2. WT, wild type.

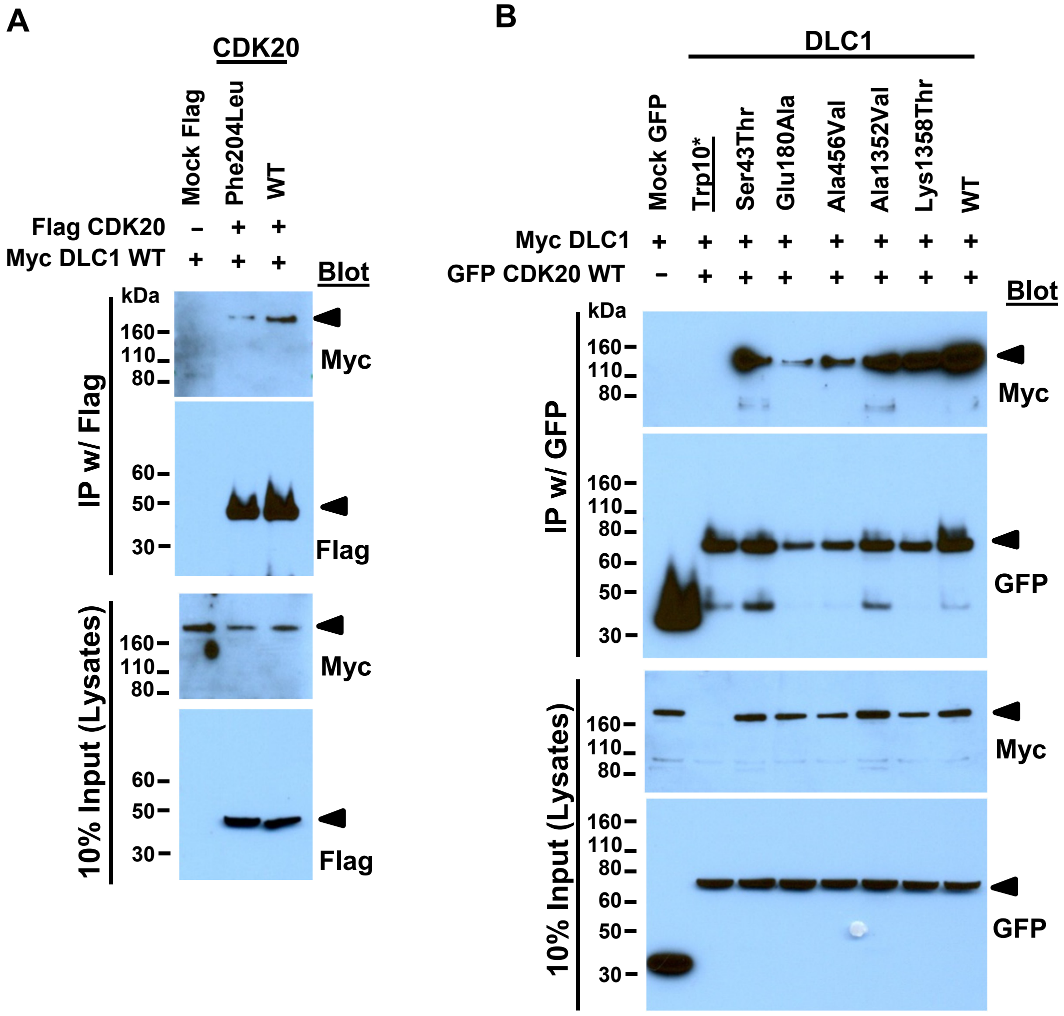

Supplementary Figure 6. CDK20 interacts with DLC1 upon co-overexpression in HEK293T cells.

(A) The mutant clone reflecting the mutation of *CDK20* identified in individual with NS does not abrogate the interaction with DLC1. This is the confirmatory experiment of **Fig. 2d**.

(B) None of the mutant clones reflecting alleles of *DLC1* identified in individuals with NS, except Arg10*, abrogate the interaction with CDK20. WT, wild type.

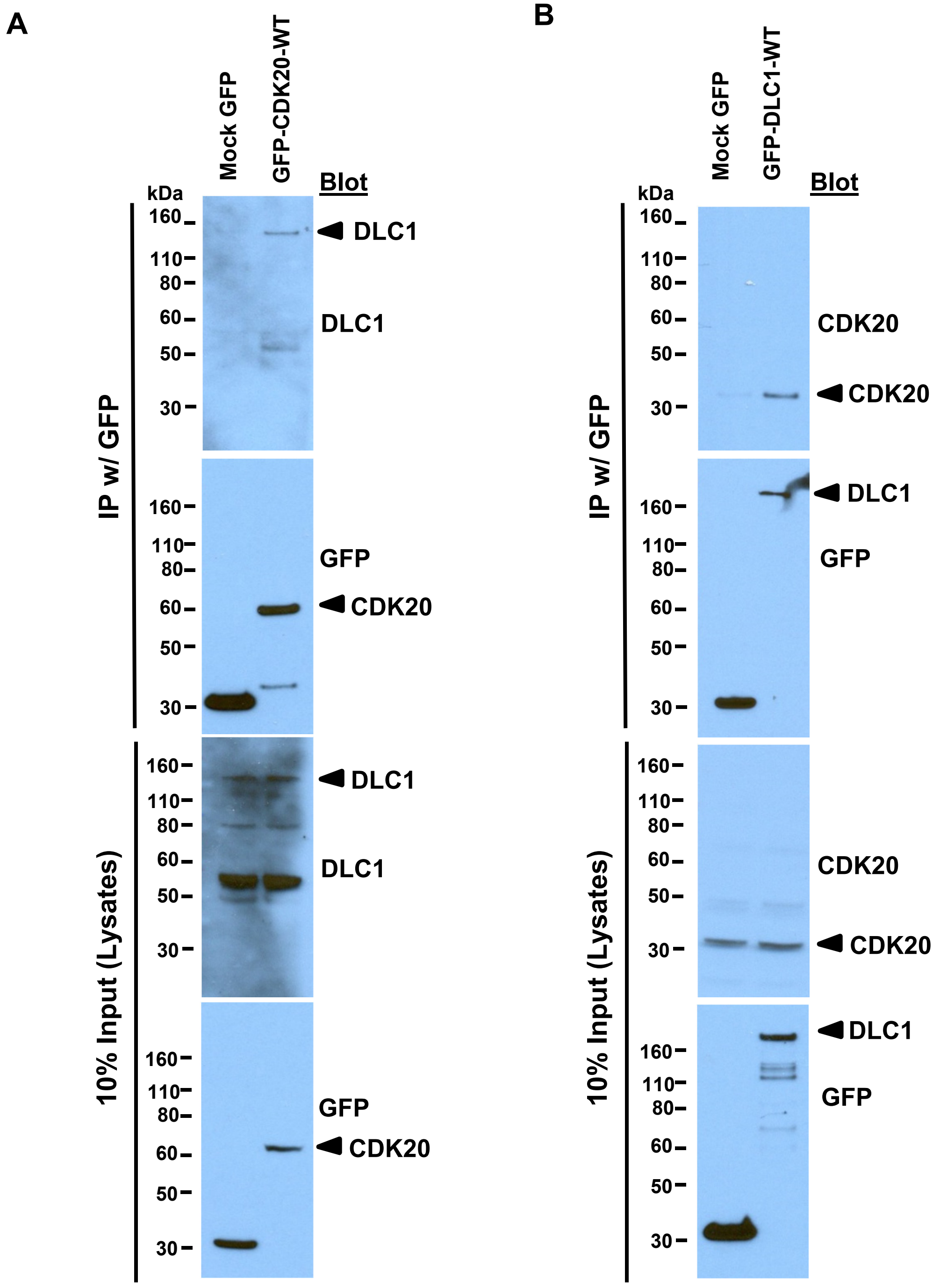

Supplementary Figure 7. CDK20 interacts with DLC1 in HEK293T cells.

A. Endogenous DLC1 interacts with overexpressed GFP-tagged human CDK20 in HEK293T cells.

B. Endogenous CDK20 interacts with overexpressed GFP-tagged human DLC1 in HEK293T cells (this is confirmatory experiment of A).

WT, wild type.

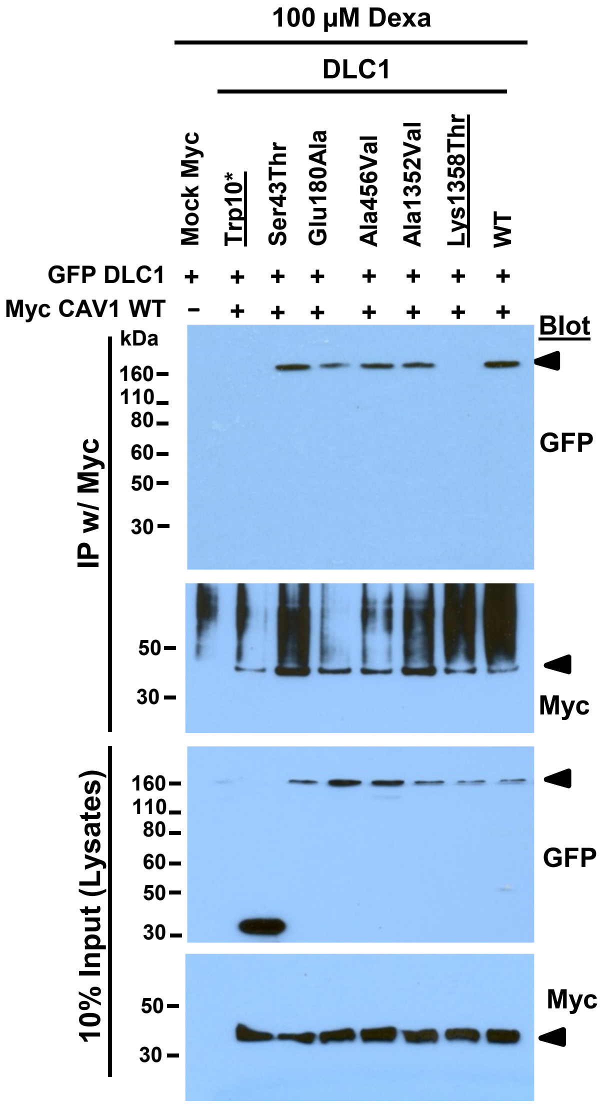

Supplementary Figure 8. Treatment with dexamethasone does not interfere with abrogation of interaction between CAV1 and DLC1 mutants Trp10* and Lys1358Thr.

CAV1 and DLC1 were co-overexpressed in HEK293T cells, which were pre- and post-treated with 100 µM of dexamethasone. However, this treatment did not interfere with abrogation of interaction of CAV1 and DLC1 by the mutants Trp10* and Lys1358Thr, as demonstrated in **Fig 2e**. Two mutant DLC1 c-DNA clones, reflecting Trp10* and Lys1358Thr alleles of NS patients A548-21 and A4967-21 respectively, still lacked this interaction. This is also a confirmatory experiment to **Fig. 2e**.

WT, wild type.

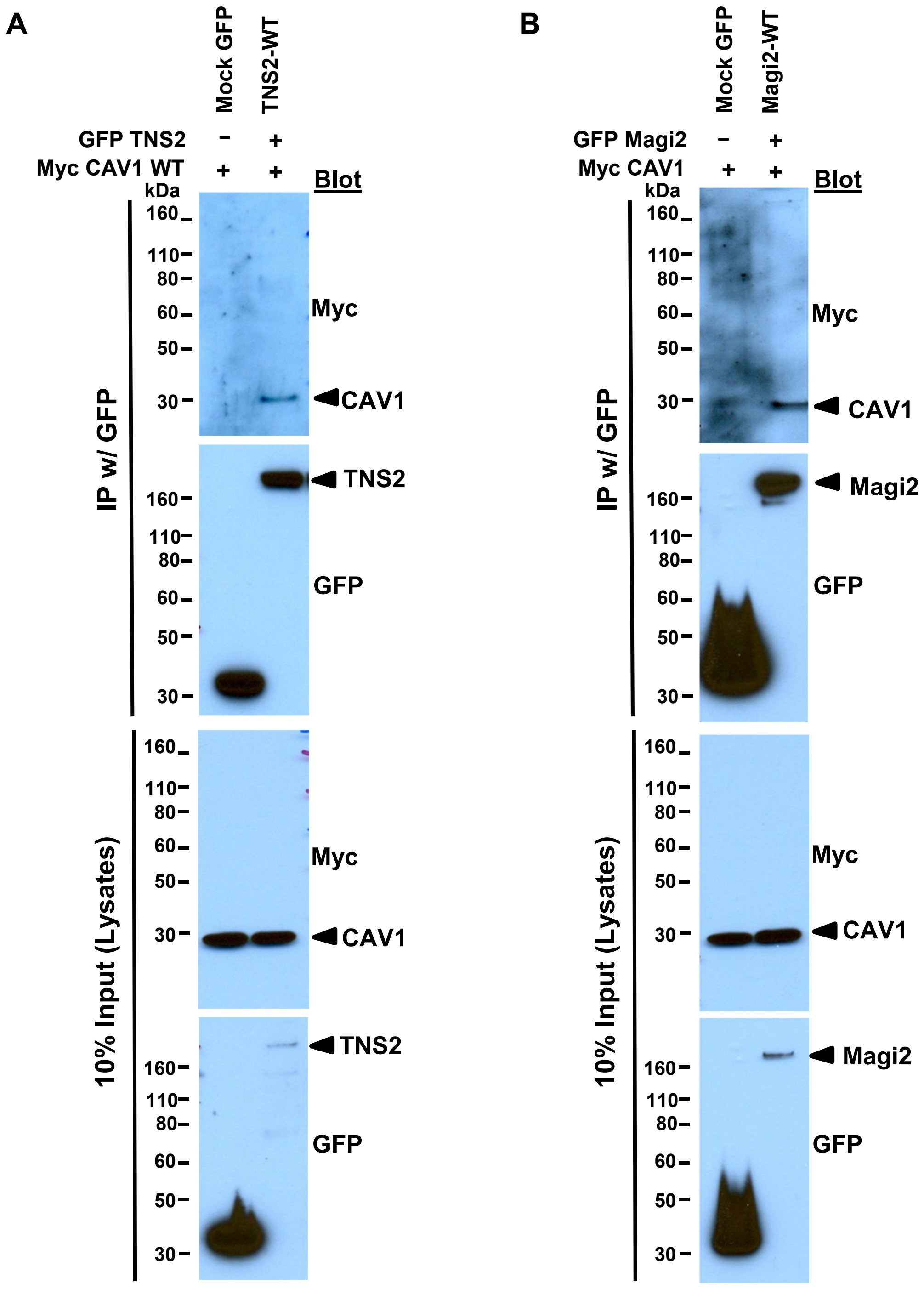

Supplementary Figure 9. GFP tagged human TNS2 (A) and GFP tagged rat Magi2 (B) interact with Myc tagged CAV1 upon co-overexpression in HEK293T cells.

WT, wild type.

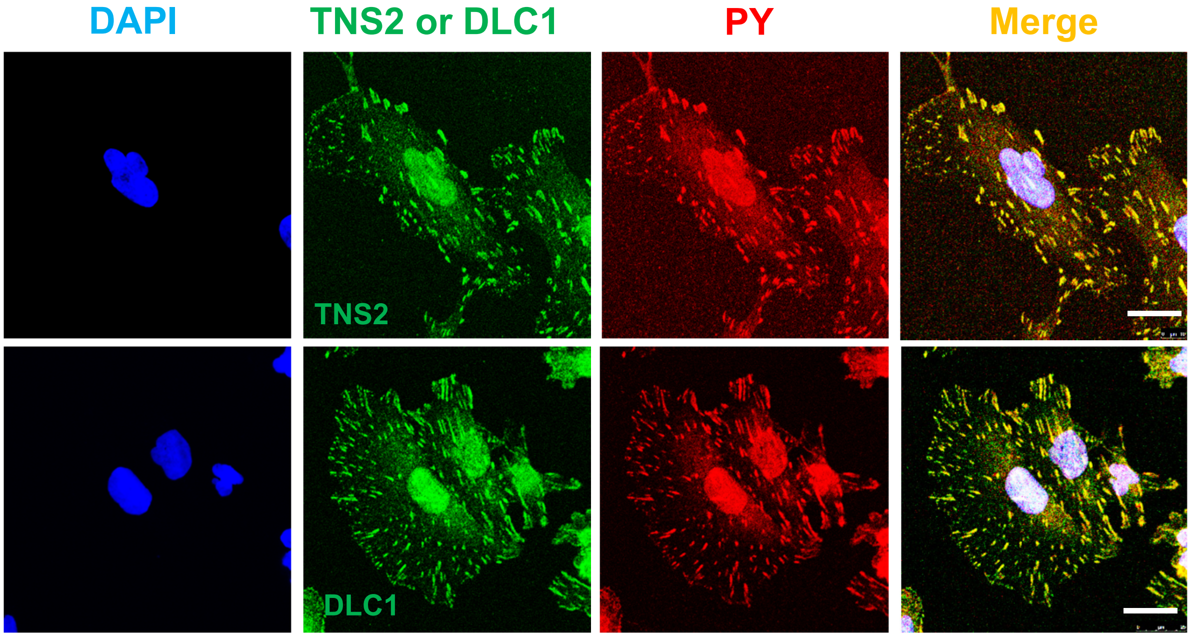

Supplementary Figure 10. Endogenous expression of TNS2 and DLC1 in cultured human podocytes.

Immortalized human podocytes were seeded on cover-glasses, and immunostained with TNS2 (green), DLC1 (green), phospho-tyrosine (PY, red) and DAPI after SDS permeabilization. TNS2, and DLC1 colocalize with phosphotyrosine at the focal adhesions in human podocytes (Scale bar 10μm).

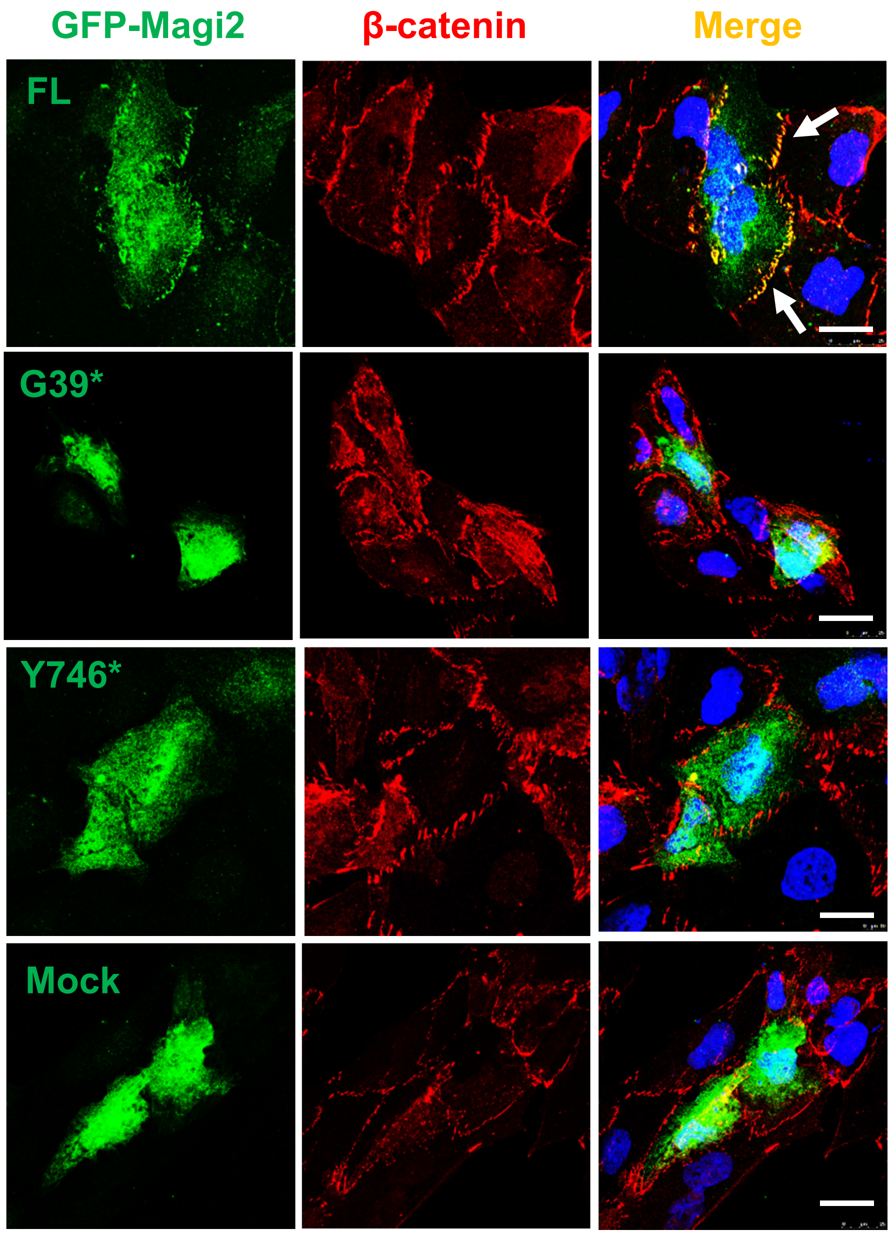

Supplementary Figure 11. Mislocalization of overexpressed MAGI2 mutant alleles at adherens junctions in human podocytes.

Upon overexpression, full length (FL) GFP-MAGI2 colocalizes with β-catenin at adherens junctions. However, overexpression of GFP-MAGI2 mutants Gly39* and Tyr746*, representing human disease alleles, fail to localize at adherens junctions (Scale bar 10µm).

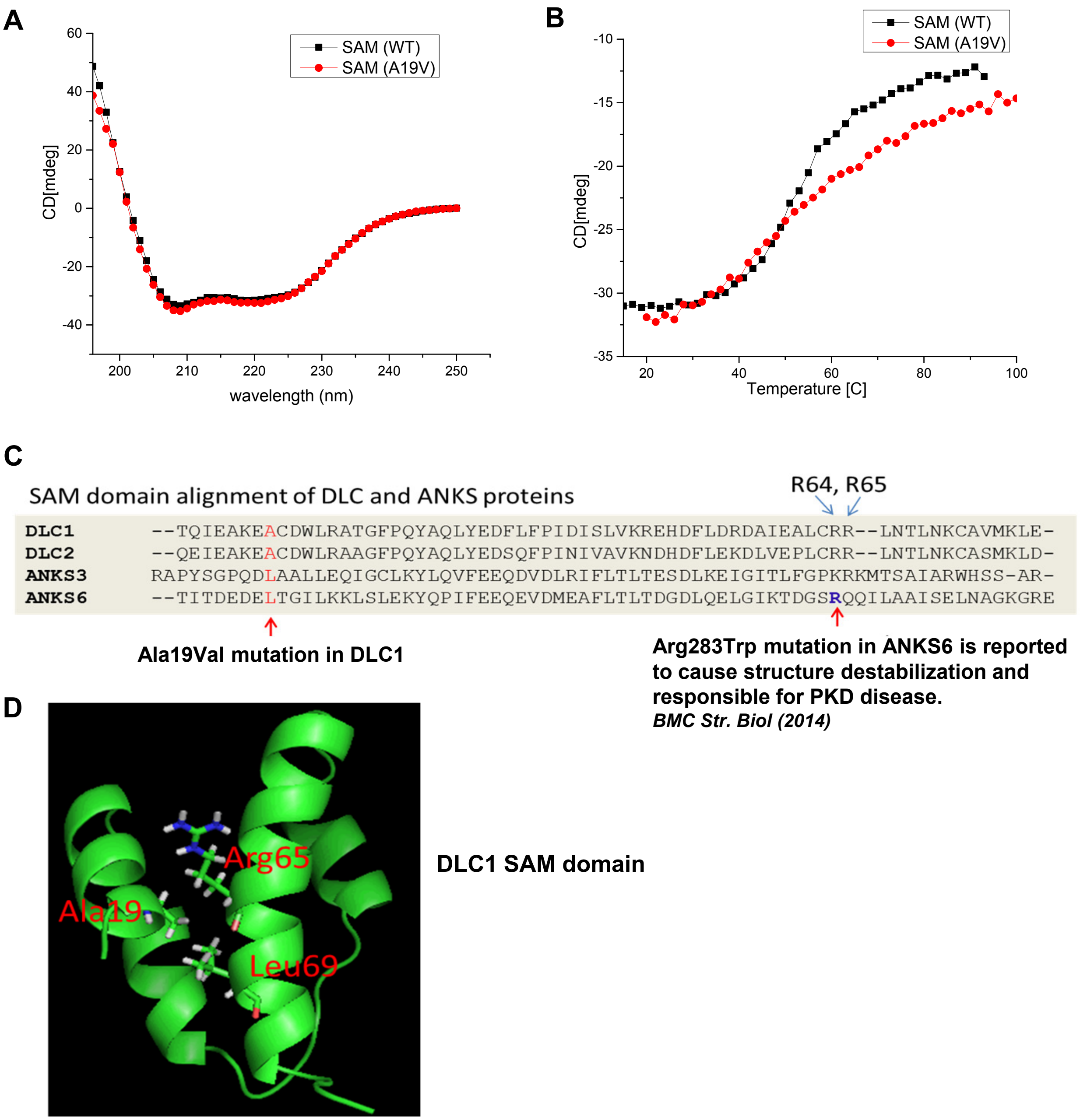

Supplementary Figure 12. Circular Dichroism modelling depicts that Ala19Val (or Ala456Val in human protein) mutant in SAM domain of DLC1 have an effect on its polymerization.

(A) CD spectra of WT and A19V SAM domain before melting.

(B) Thermal denaturation curves of wild type (WT) and Ala19Val SAM domains. The denaturation temperature of WT SAM domain was near to 58°C and the melting curve was sigmoidal indicating cooperative unfolding behavior. However, for A19V SAM, there was loss of cooperativity and absence of a sharp melting point.

(C-D) Arg823Trp mutation in ANKS6 (Arg65) and Ala456Val mutation in DLC1 (Ala19) are located in proximity in DLC1-SAM domain structure indicating a possible link of similar destabilization effect.

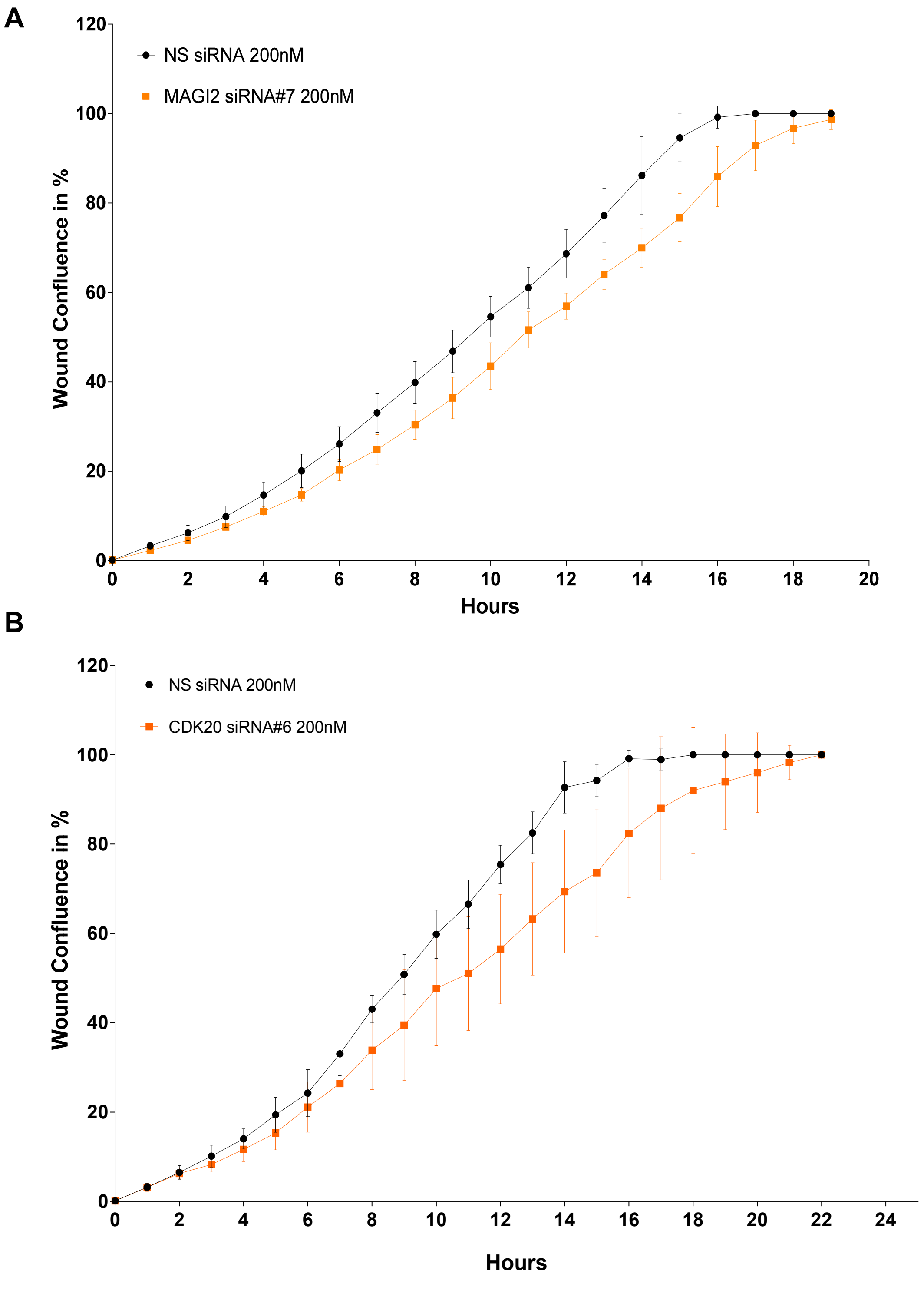

Supplementary Figure 13. Transient knockdown of *MAGI2* or *CDK20* decreases cell migration in cultured human podocytes.

siRNA mediated knockdown of *MAGI2* **(A)** or *CDK20* **(B)** leads to decreased podocyte migration compared with those transfected with scrambled siRNA (NS). Error bars represent SD for at least 3 independent experiments (linear regression with slope difference analysis). Migration assay was performed using the IncuCyte® system (described in Methods).

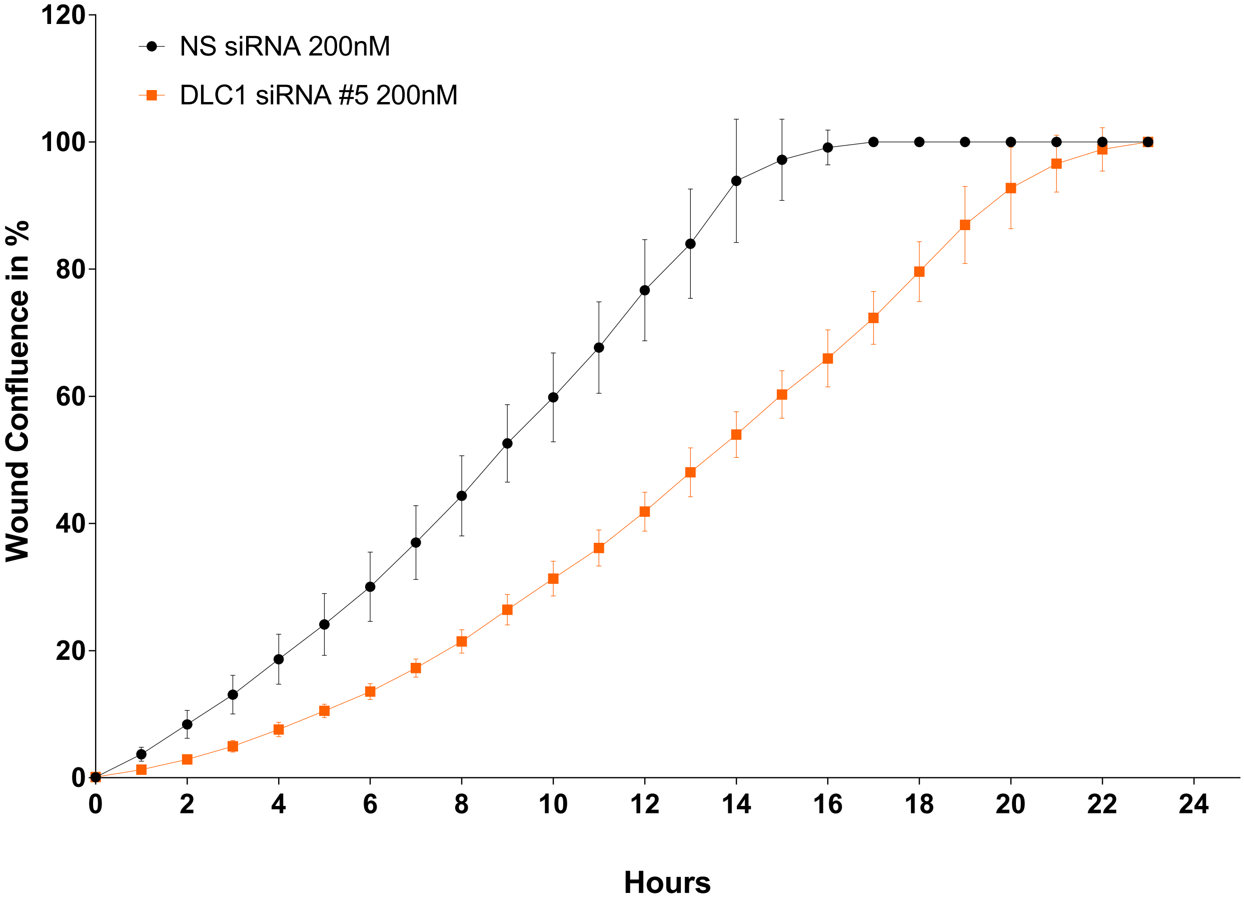

Supplementary Figure 14. Transient knockdown of *DLC1* decreases cell migration in cultured human podocytes.

siRNA mediated knockdown of *DLC1* leads to decreased podocyte migration compared with those transfected with scrambled siRNA (NS). Error bars represent SD for at least 3 independent experiments (linear regression with slope difference analysis). Migration assay was performed using the IncuCyte® system (described in Methods).

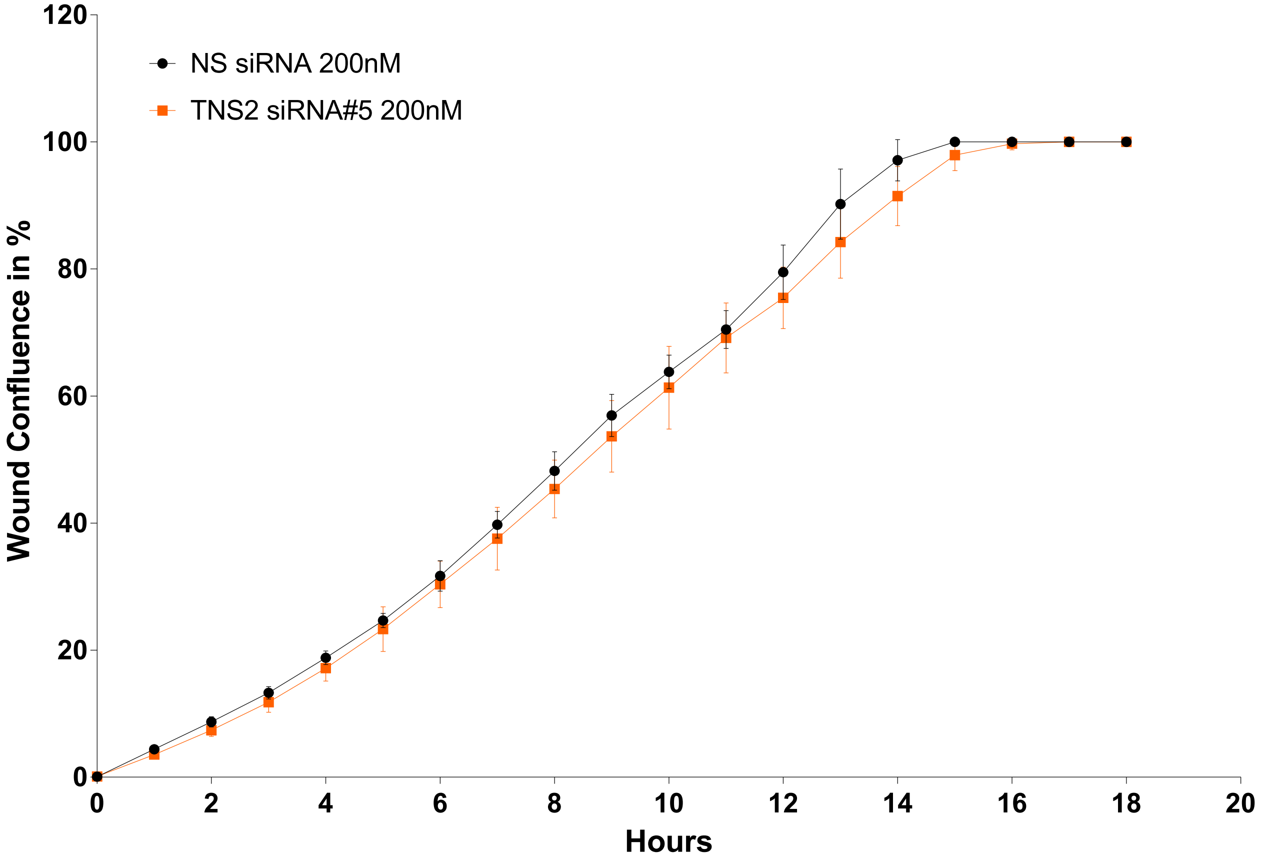

Supplementary Figure 15. Transient knockdown of *TNS2* does not affect podocyte migration.

Transient knockdown of *TNS2* did not show any change in podocytes migration compared with cells transfected with scrambled siRNA (NS). Error bars represent SD for at least 3 independent experiments (linear regression with slope difference analysis). Migration assay was performed using the IncuCyte® system (described in Methods).

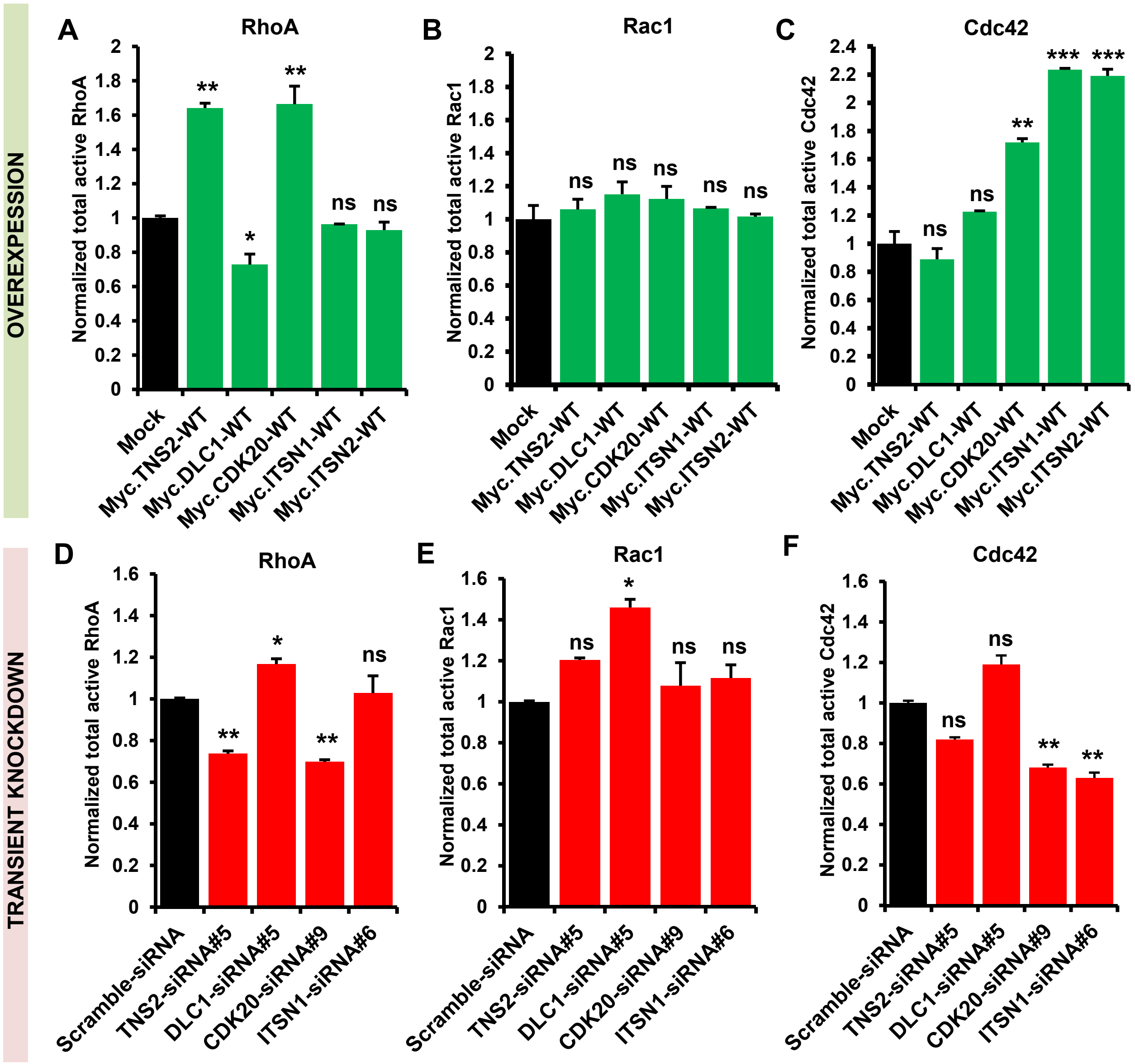

Supplementary Figure 16. Effect of overexpression and siRNA mediated knockdown of *TNS2*, *DLC1*, *CDK20* and *ITSN* on active RhoA, Rac1 or Cdc42 in HEK293T cells, as measured by the G-LISA assay.

(**A-C**) Myc-tagged *TNS2*, *DLC1*, *CDK20*, *ITSN1* or *ITSN2* constructs were overexpressed in HEK293T cells, and levels of active RhoA (**A**), Rac1 (**B**) or Cdc42 (**C**) were monitored using the G-LISA assay. T-test was performed versus Mock-Myc (Mock) treated control. Note that the overexpression of *TNS2, DLC1* or *CDK20* affects active RhoA (also shown in **Fig 3b-d**) while the overexpression of *CDK20, ITSN1* or *ITSN2* affects active CDC42.

(**D-F**) siRNA mediated knockdown was performed for *TNS2*, *DLC1*, *CDK20*, or *ITSN1* in HEK293T cells, and levels of active RhoA (**D**), Rac1 (**E**) or Cdc42 (**F**) were measured using the G-LISA assay. T-test was performed versus scrambled-siRNA treated control. Note that knockdown of *TNS2, DLC1* or *CDK20* affects active RhoA (also shown in **Fig 3f-h**) while the knockdown of *CDK20, ITSN1* or *ITSN2* affects active CDC42. Knockdown of *DLC1* always showed significant increase in active Rac1. Error bars are defined as the standard error of at least three independent experiments. One-way ANOVA with Dunnet's post hoc test was performed either for mock control *versus* WT (**A-C**) or scramble knockdown *versus* each gene-specific siRNA (**D-F**). Data are presented as the mean ± SEM. * $P < 0.05$, ** $P < 0.01$,*** $P < 0.001$. ns, non significant.

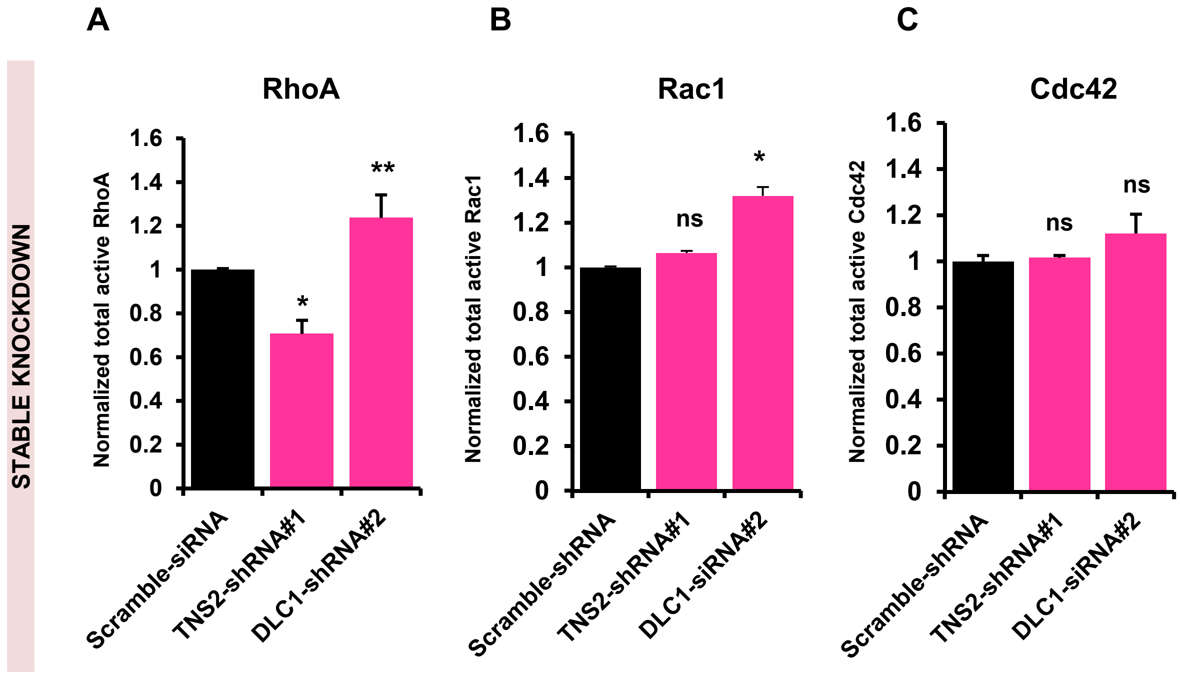

Supplementary Figure 17. Effect of stable knockdown of *TNS2* and *DLC1* on active RhoA, Rac1 or Cdc42 in HEK293T cells, as measured by the G-LISA assay.

(**A-C**) shRNA-mediated knockdown was performed for *TNS2* or *DLC1* in HEK293T cells, and levels of active RhoA (**A**), Rac1 (**B**) or Cdc42 (**C**) were measured using the G-LISA assay. T-test was performed versus scrambled-siRNA treated control. Note that knockdown of *TNS2* decreases active RhoA and knockdown of *DLC1* increases active RhoA (also shown in **Supplementary Fig. 16**). Knockdown of *DLC1* showed a significant increase in active Rac1.

Error bars are defined as the standard error of at least three independent experiments. One-way ANOVA with Dunnet's post hoc test was performed for scramble control *versus* TNS2-shRNA#1 or DLC-shRNA#2. Data are presented as the mean ± SEM. * $P < 0.05$, ** $P < 0.01$. ns, non significant.

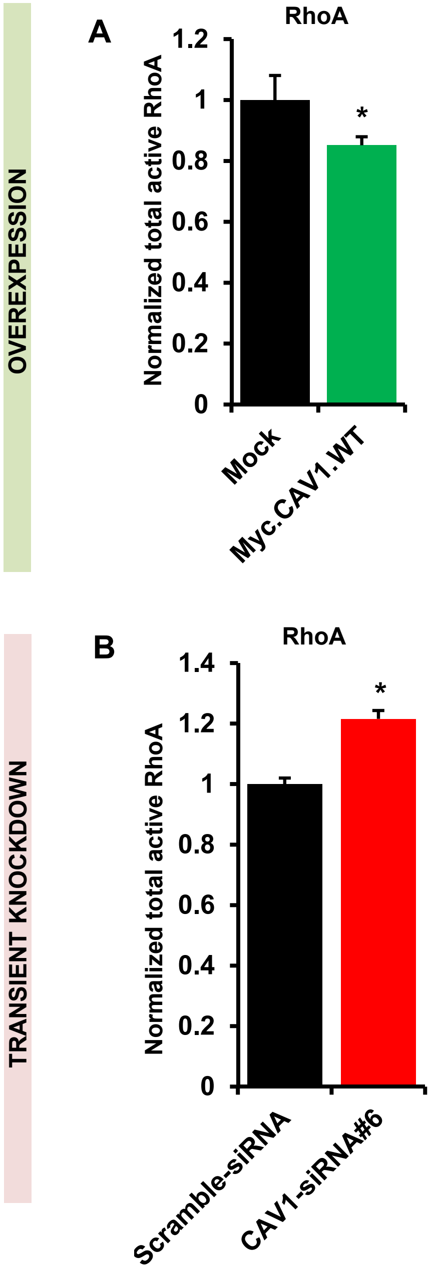

Supplementary Figure 18. Effect of overexpression and siRNA mediated knockdown of *CAV1* on active RhoA in HEK293T cells, as measured using the G-LISA assay.

(A) Myc-tagged *CAV1* construct was overexpressed in HEK293T cells, and levels of active RhoA were measured using the G-LISA assay. One-way ANOVA with Dunnet's post hoc test was performed for mock control *versus* CAV1-WT.

(B) siRNA-mediated knockdown was performed for *CAV1* in HEK293T cells, and levels of active RhoA were measured using the G-LISA assay. One-way ANOVA with Dunnet's post hoc test was performed for scramble control *versus* CAV1-siRNA#6.* $P < 0.05$. Error bars are defined as the standard error of at least three independent experiments.

Note that the overexpression of *CAV1* decreased active RhoA, whereas the knockdown of *CAV1* increased active RhoA.

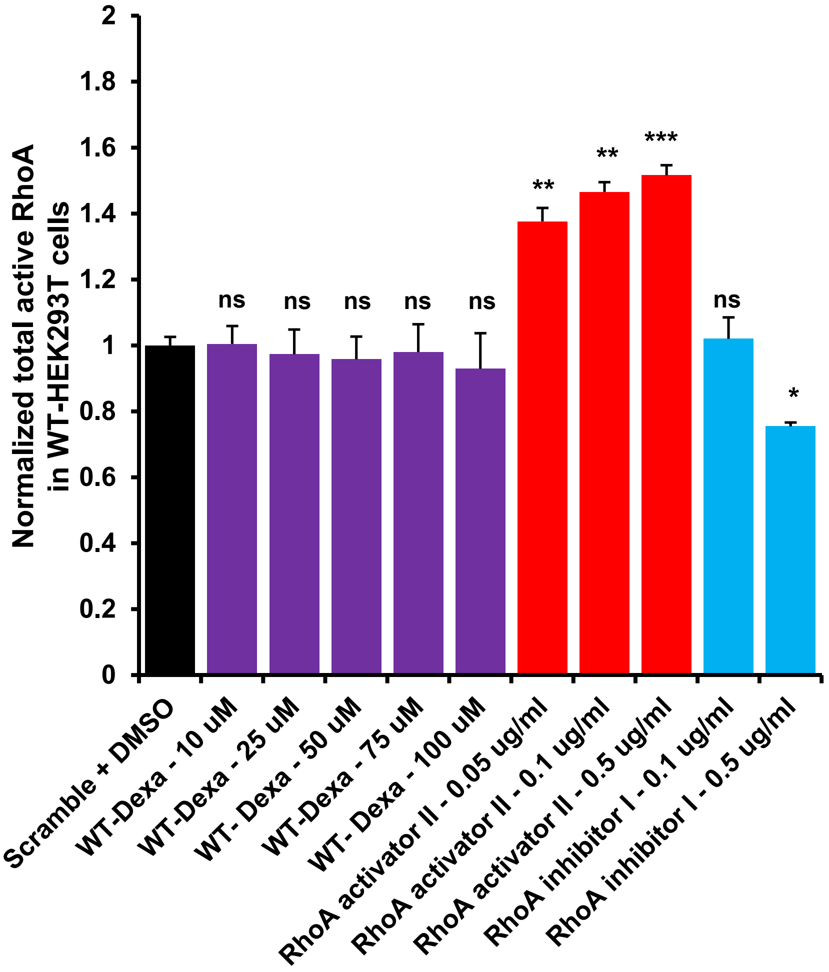

Supplementary Figure 19. Effect of different concentrations of dexamethasone on wild type HEK293T cells as measured using the G-LISA assay.

Wild type HEK293T cells were treated with different dosages of dexamethasone (Dexa) (10μM, 25μM, 50μM, 75μM and 100μM) and levels of active RhoA were measured using the G-LISA assay. One-way ANOVA with Dunnet's post hoc test was performed *versus* DMSO (10μM) treated control. * $P < 0.05$, ** $P < 0.01$, *** $P < 0.001$. ns, non significant; WT, wild type.

Treatments with RhoA activators (red) and RhoA inhibitors (blue) were used as positive controls.

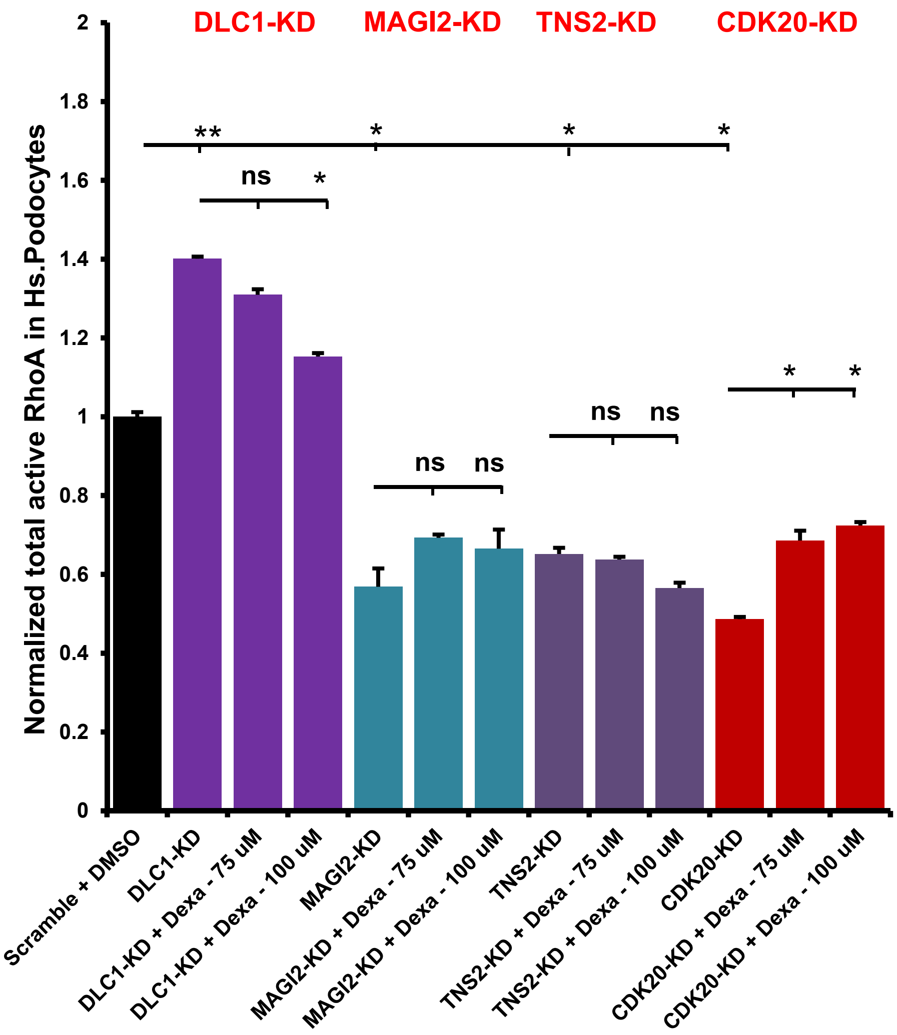

Supplementary Figure 20. Effect of two different concentrations of dexamethasone (75 μM and 100 μM) on RhoA activation upon knockdown of *DLC1, MAGI2, TNS2* or *CDK20* in human podocytes, as measured using the G-LISA assay.

The change in active RhoA upon knockdown of *DLC1* or *CDK20*, but not *MAGI2,* or *TNS2* was found reversible by treatment of human podocytes with 100 μM dexamethasone. Error bars are defined as the standard error of at least three independent experiments. One-way ANOVA with Dunnet's post hoc test was performed *versus* DMSO (10 μM) treated control. Data are presented as the mean ± SEM. * $P < 0.05$, ** $P < 0.01$. ns, non significant.

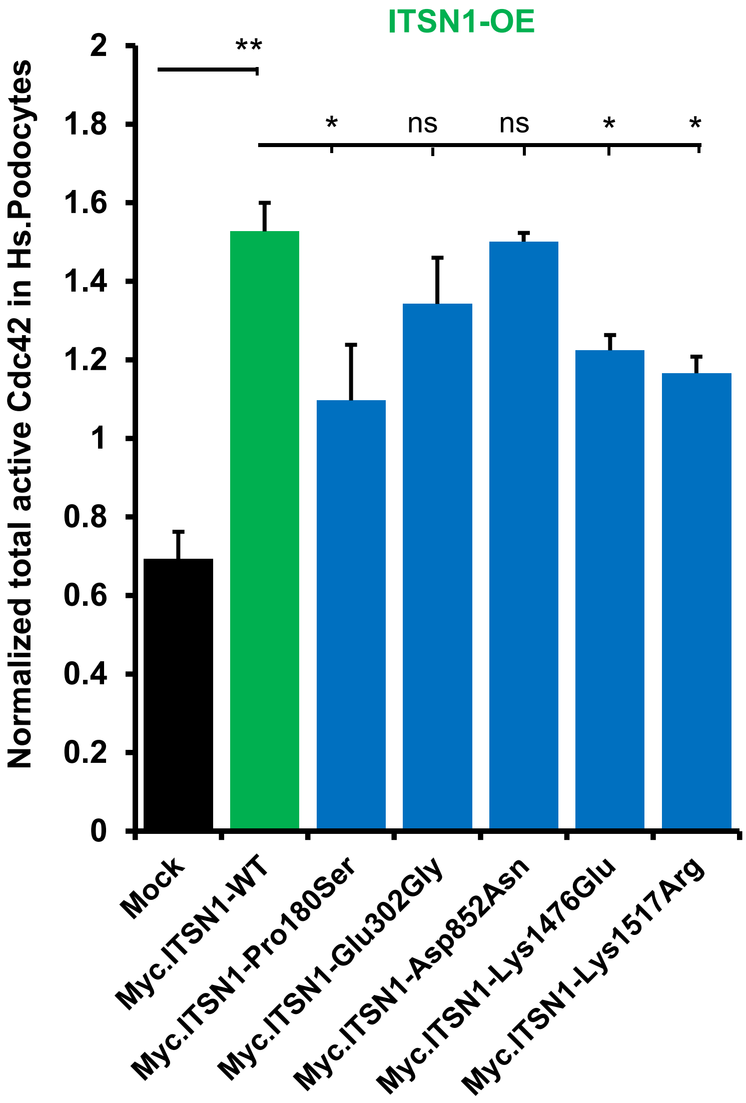

Supplementary Figure 21. Effect of overexpression in *ITSN1,* wild type (WT) and mutants, on Cdc42 activation in human podocytes, as measured using the G-LISA assay.

Myc-tagged *ITSN1*-WT increased active Cdc42 levels upon overexpression in human podocyte as compared to mock control. However, 3 out of 5 *ITSN1* mutants from pTSNS patients failed to show any significant increase in active Cdc42 upon overexpression. One-way ANOVA with Dunnet's post hoc test was performed *versus* mock control or ITSN1-WT. Data are presented as the mean ± SEM. * $P < 0.05$, ** $P < 0.01$. ns, non significant.

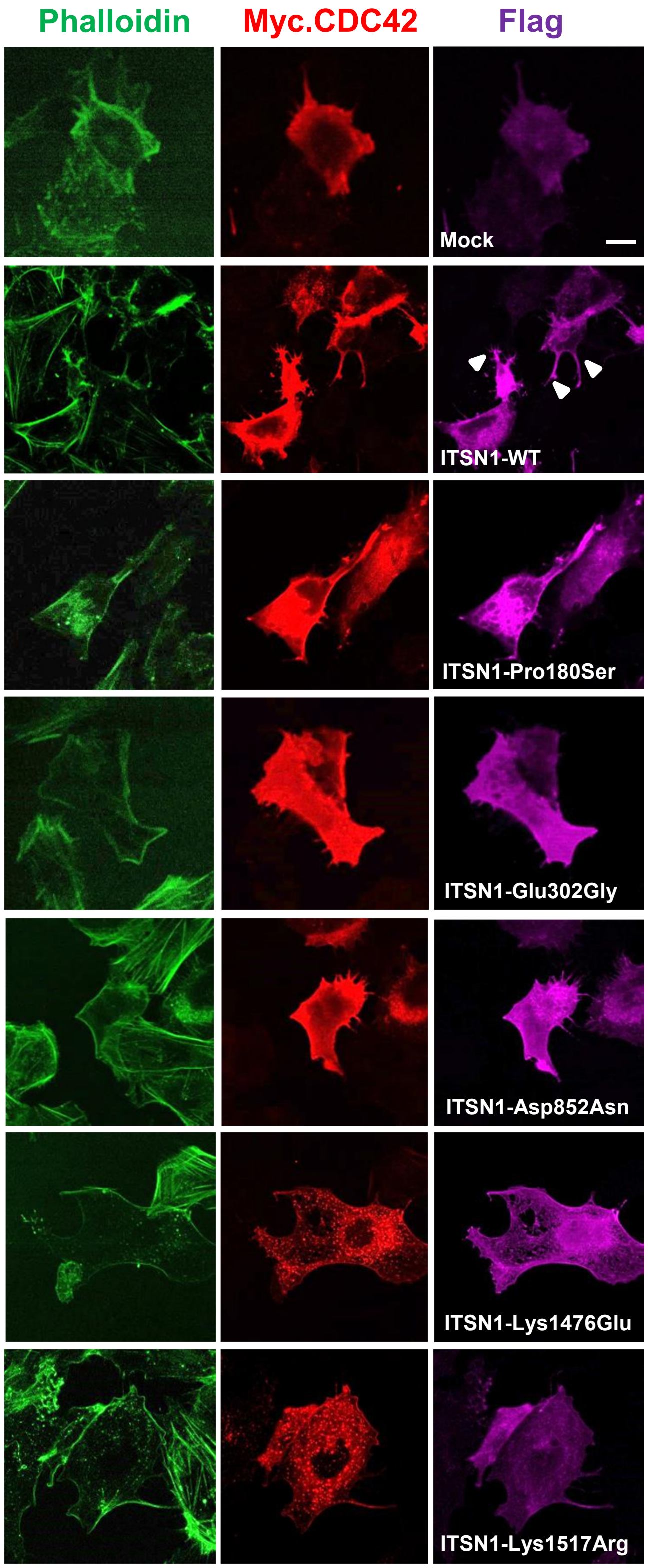

Supplementary Figure 22. Filopodia induction in cultured human podocytes upon overexpression of *ITSN1,* wild type (WT) and mutants.

Human podocytes were transfected with Myc-Cdc42 and FLAG tagged WT *ITSN1*, 5 mutants (Pro180Ser, Glu302Gly, Asp852Asn, Lys1476Glu and Lys1517Arg) or mock-transfected. Cells were stained with Phalloidin (green), anti-Myc antibodies (red) or anti-FLAG antibodies (pink). Immunofluorescence images showed extensive filopodia induction in WT *ITSN1*-L-transfected cells (white arrowheads) whereas overexpression of ITSN2 mutants representing mutations in patients with pTSNS lacked strong filopodia formation. Representative images are shown. (Scale bar 50µm).

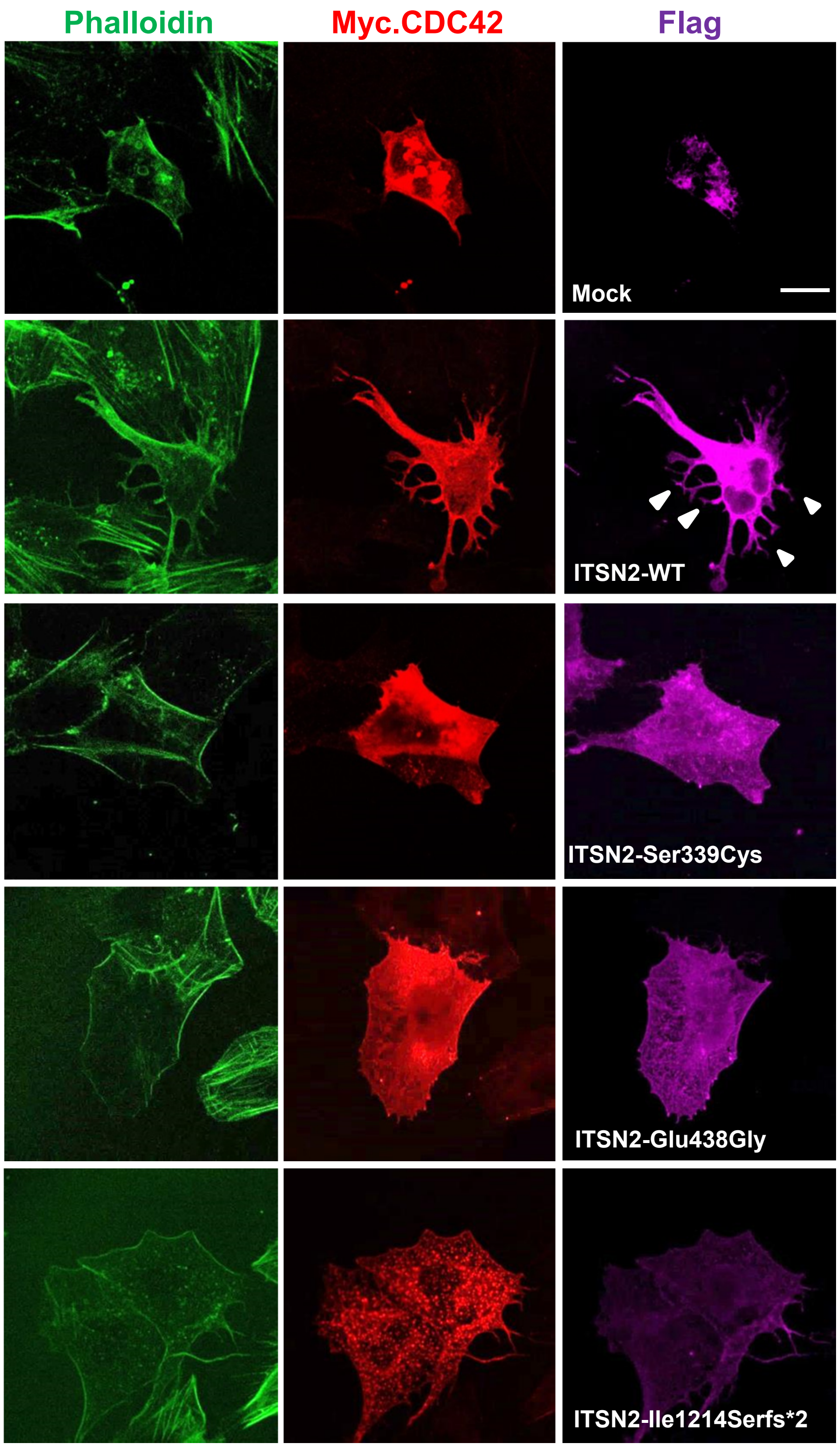

Supplementary Figure 23. Filopodia induction in cultured human podocytes upon overexpression of *ITSN2,* wild type (WT) and mutants.

Human podocytes were transfected with Myc-Cdc42 and FLAG tagged WT *ITSN2*-L, or 3 mutants from patients with pTSNS (Ser339Cys, Glu438Gly and Ile1214Serfs*2) or mock-transfected. Cells were stained with Phalloidin (green), anti-Myc antibodies (red), or anti-FLAG antibodies (pink). Immunofluorescence images showed extensive filopodia induction in WT *ITSN2*-L-transfected cells (white arrowheads). Representative images are shown (Scale bar 50μm).

A

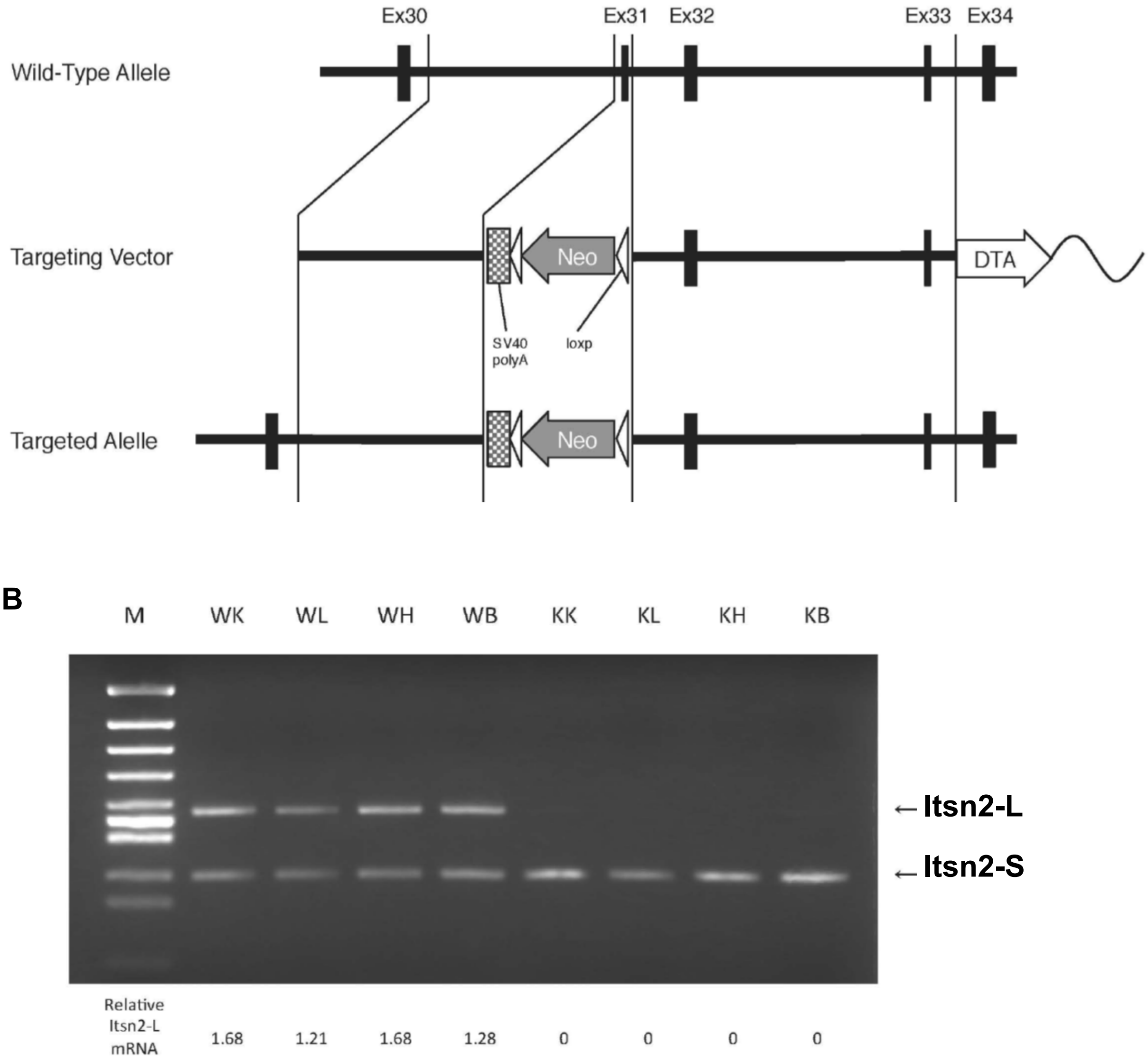

Supplementary Figure 24. Generation of Itsn2-L knockout (KO) mice.

(A) The targeting vector was designed to generate a conventional KO mouse.

(B) Itsn2 mRNA of various organs were analyzed by RT-PCR. Reverse primers were designed for Itsn2 short (Itsn2-S) and long (Itsn2-L). The bands of Itsn2-L were not detected in *Itsn2* $^{L-/L-}$ mice. The mRNA levels of Itsn2-L relative to those of Itsn2-S are shown.

M, size marker; WK, WT kidney; WL, WT liver; WH, WT heart; WB, WT brain; KK, KO kidney; KL, KO liver; KH, KO heart; KB, KO brain.

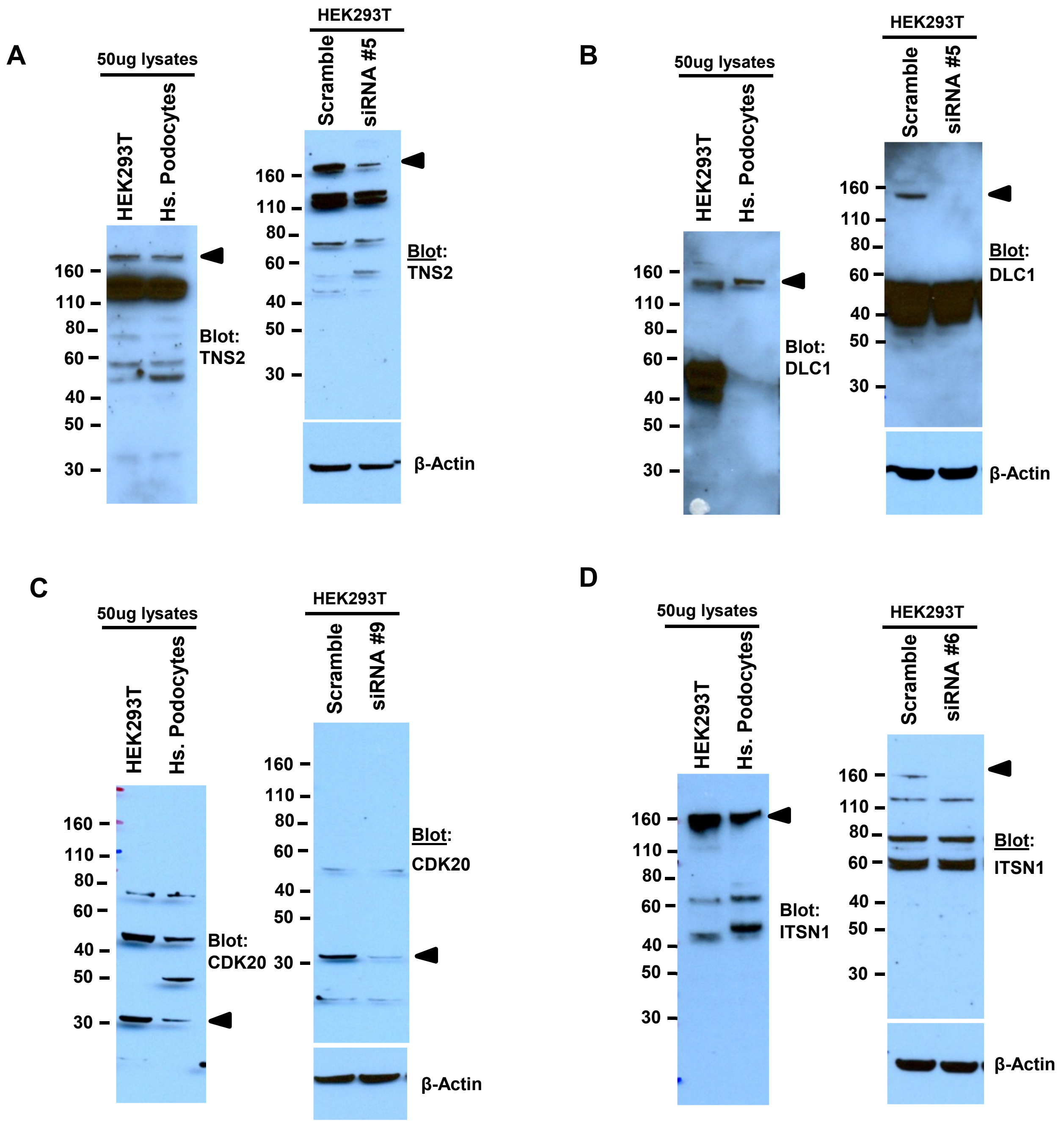

Supplementary Figure 25. Characterization of antibodies against TNS2 (A), DLC1 (B), CDK20 (C) and ITSN1 (D).

Left panels: Endogenous expression of each protein in 50 µg of HEK293T and human podocyte cell lysates. Arrow heads indicate the relative positions of the respective proteins.

Right panels: siRNA-mediated knockdowns in HEK293T cells confirm the specificity of four antibodies against TNS2, DLC1, CDK20, and ITSN1 respectively in HEK293T cells. Arrow heads indicate the relative positions of the respective protein. (TNS2: Hafizi S. *et al.* 2005; DLC1, Li G. *et al*. 2011; CDK20, Novus-NBP1-91214, rabbit polyclonal; ITSN1, abcam-ab118262 rabbit polyclonal)

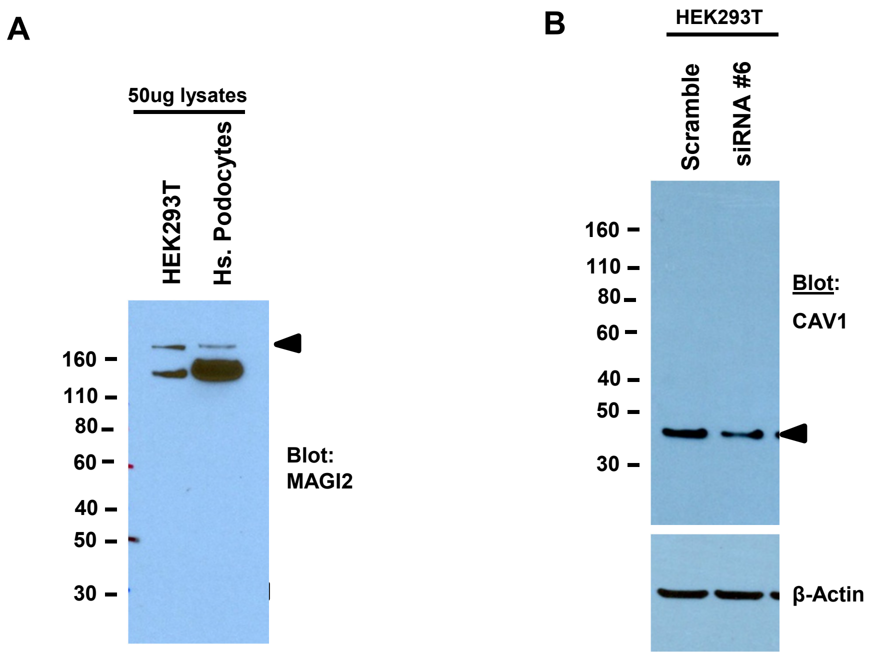

Supplementary Figure 26. Characterization of antibodies against MAGI2 and CAV1.

(A) Endogenous expression of MAGI2 in 50 μg of HEK293T and human podocyte cell lysates. Arrow head indicate the expected size of the protein.

(MAGI2- Gee HY et al; 2009)

(B) siRNA-mediated knockdown in HEK293T cells confirms the specificity of antibody against CAV1. Arrow head indicate the relative size of the protein.
(CAV1: Cell Signaling, rabbit polyclonal)

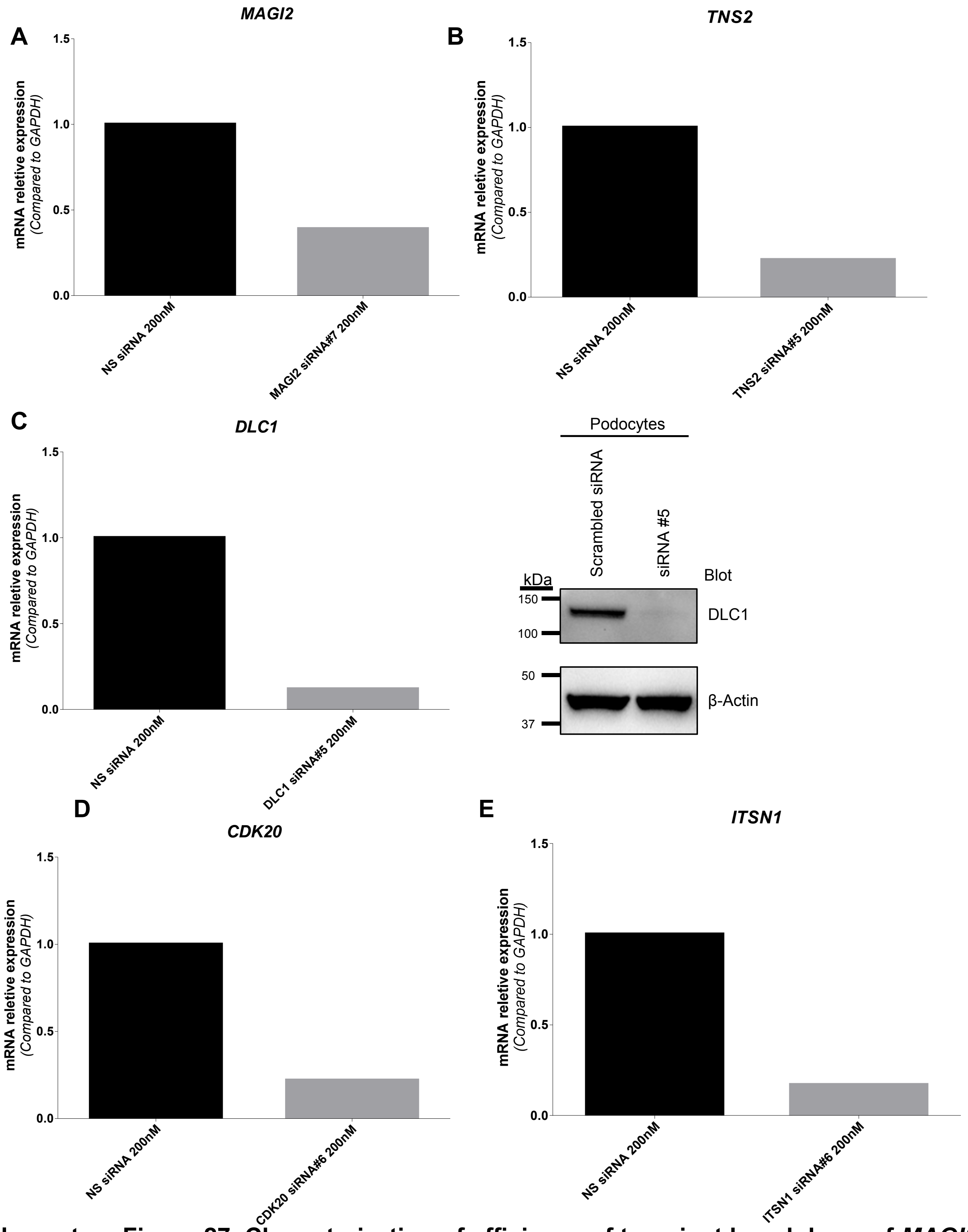

Supplementary Figure 27. Characterization of efficiency of transient knockdown of *MAGI2* (A), *TNS2* (B), *DLC1* (C), *CDK20* (D) and *ITSN1* (E) in undifferentiated human podocytes.
mRNA levels were measured by real-time PCR using TaqMan probes for *MAGI2* **(A)**, *TNS2* **(B)**, *DLC1* **(C)**, *CDK20* **(D)**, and *ITSN1* **(E)**, respectively, with *GAPDH* as control. Knockdown of DLC1 was also confirmed by western blotting with antibody against *DLC1* **(C)**.

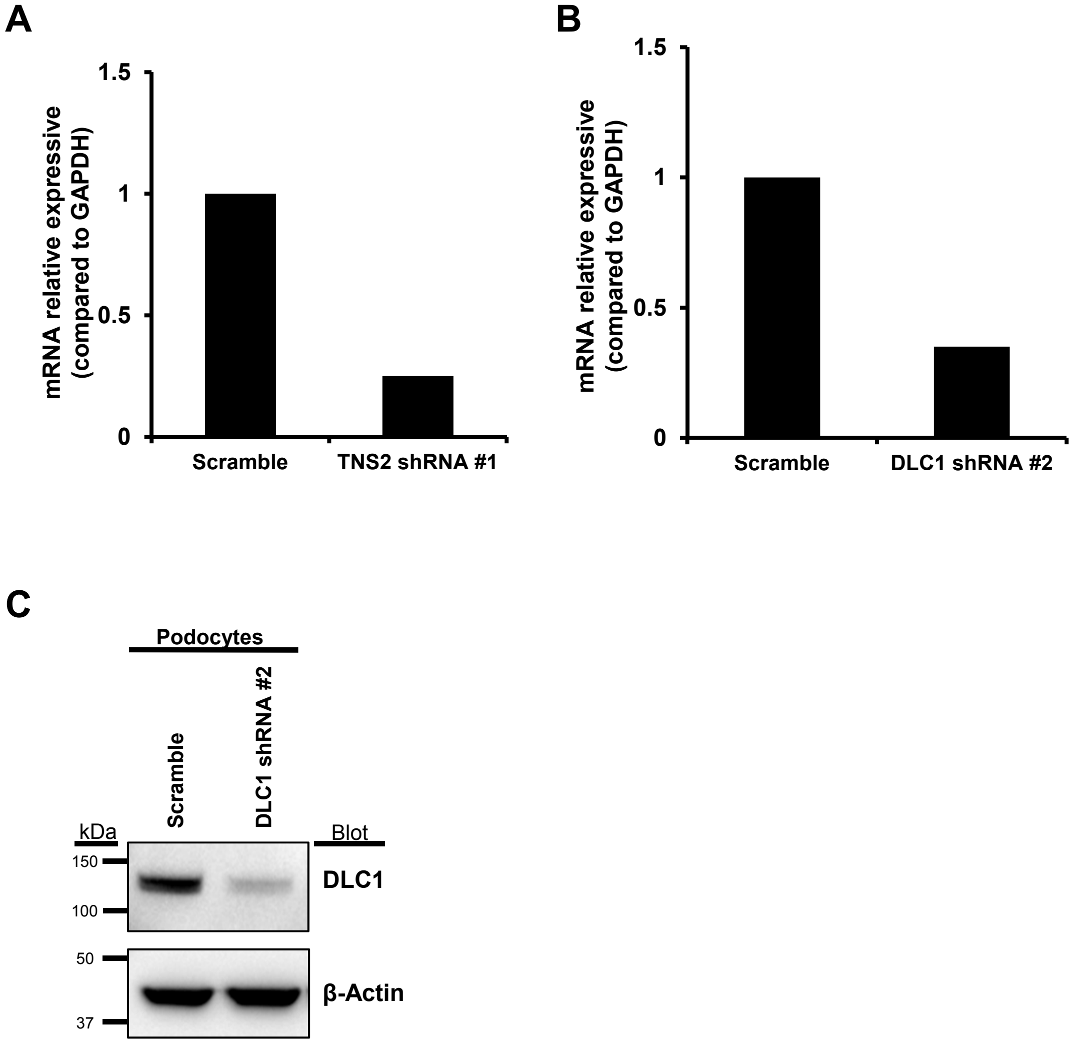

Supplementary Figure 28. Characterization of efficiency of shRNA-mediated stable knockdown of *TNS2* and *DLC1* in human podocytes.

(A-B) mRNA levels were measured by real-time PCR using TaqMan probes for *TNS2* **(A)**, and *DLC1* **(B)**, respectively, with *GAPDH* as control.

(C) Knockdown of DLC1 was also confirmed by western blotting with antibody against *DLC1*.

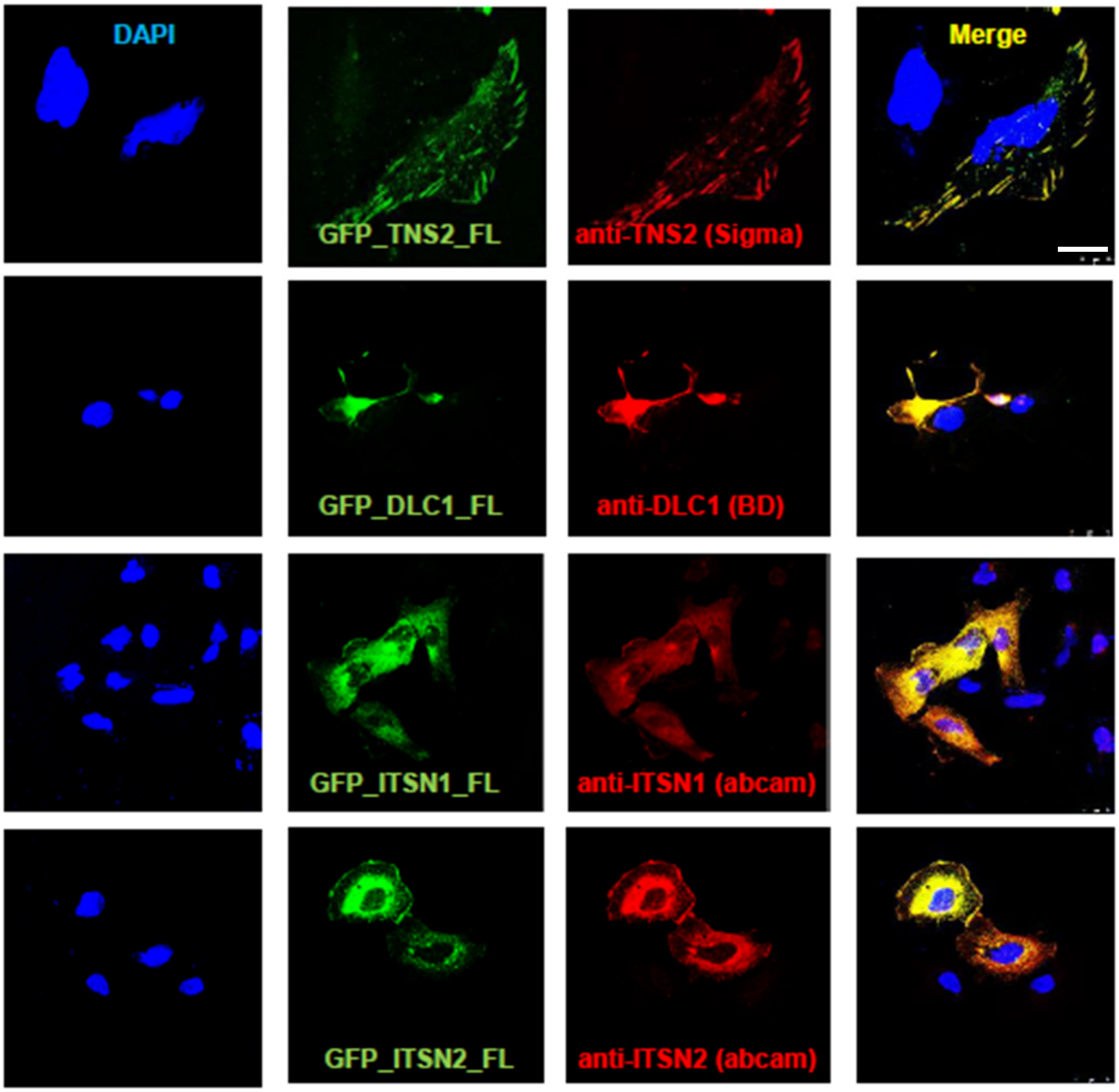

Supplementary Figure 29. Characterization of antibodies against TNS2, DLC1, ITSN1 and ITSN2 with immunofluorescence.

Immortalized human podocytes were seeded on cover-glasses and transfected with 4ug of GFP tagged full length *TNS2, DLC1, ITSN1, ITSN2, CDK20*, and *CAV1*. 24 hours post transfection cells were stained with the respective antibodies (Scale bar 10µm).

Anti-TNS2 (Sigma, HPA034659), anti-DLC1 (BD Clone 3/DLC-1), anti-ITSN1 (abcam ab118262), anti-ITSN2 (abcam ab176592).

Supplementary Table 1: Recessive mutations detected in genes *MAGI2, TNS2, DLC1, CDK20, ITSN1* and *ITSN2* in individuals with NS.

Family -Individual	Ethnic origin	Parental consan-guinity	Nucleotide alteration (s)	Alteration(s) in coding sequence [a]	Exon (segrega-tion)	Amino acid sequence conservation	GnomAD Allele count (zygosity)	Age at Onset	Kidney disease	Age at ESKD	Treatment and renal transplantation	Histology (at age)	Extrarenal manifestations
MAGI2													
A5146 -21	Arab (Iraq)	Yes	c.115G>T	p.Gly39*	1 (HOM, M, P)	NA	NP	4 yr	SSNS	ND	partial response to Pred/CsA	FSGS (4 yr)	Hydrocephalus
B91 (1302/11)	Europe	No	c.2238C>G	p.Tyr746*	12 (HOM, maternal isodisomy)	NA	NP	8 wks	CNS	ND	ND	FSGS (20 mo)	Microcephaly, severe global developmental delay, cryptorchidism, hypothyroidism, gastroesophageal reflux (IUGR and postnatal short stature - probably attributable to matUPD7)
TNS2													
A1358 -21	Turkey	Yes	c.875G>A	p.Arg292Gln	11 (HOM, ND)	*C. intestinalis*	NP	7 yr	NS	ND	no treatment attempt	MCNS (7 yr)	Asthma
A283 -21	Europe	No	c.1334G>A<br>c.3382A>G	p.Arg445Gln<br>p.Thr1128Ala	17 (het, M)<br>20 (het, P)	*D. rerio*<br>*G. gallus*	68 / 277,108 (het)<br>1522/ 240,618 (het)	7 yr	SDNS	ND	partial response to CsA; complete remission to MMF (no relapse during 2 years)	MCNS (7 yr)	ND
2605	Nigeria	No	c.1675G>A	p. Gly559Arg	17 (HOM, ND)	*D. rerio*	34 / 160,586 (het)	ND	SSNS	ND	partial response to Pred	FSGS (ND)	Hypertension
A3775 -21	India	No	c.2574C>G	p.Ile858Met	18 (HOM, ND)	*C. elegans*	186 / 232,392 (het)	2 yr	SSNS	ND	complete, remission with steroids	DMS (2 yr)	ND
A1640 -21	India	No	c.1693C>T<br>c.2574C>G	p.Arg565Trp<br>p.Ile858Met	18 (het, P)<br>18 (het, M)	*D. rerio*<br>*C. elegans*	22/ 119,318 (het)<br>186 / 232,392 (het)	3 yr	SDNS	ND	complete remission to CsA, MMF, CTX and RTX	MCNS (3 yr)	Short stature
DLC1													
A548 -21	Europe	No	c.29G>A<br>c.128G>C	p.Trp10*<br>p.Ser43Thr	2 (het, ND)<br>2 (het, ND)	NA<br>*G. gallus*	NP<br>2/ 246,110 (het)	56 yr	SRNS	62 yr	Dialysis at 62 yrs and 1st transplant at 64 yrs of age!	FSGS (56 yr)	ND
A4967 -21	Arab	No	c.539A>C<br>c.4073A>C	p.Glu180Ala<br>p.Lys1358Thr	2 (het, M)<br>15 (het, P)	*G. gallus*<br>*C. intestinalis*	NP<br>NP	7 yr	SRNS	7 yr	no response to Methyl-Pred/Pred	FSGS (7 yr)	HT, seizures (mild brain atrophy with dilated ventricles), Nephritis

A3118-21	Asia	No	c.1367C>T	p.Ala456Val	6 (HOM, M, P)	*C. elegans*	NP	6 yr	SSNS	ND	complete remission (CsA) → Relapse → MMF+CsA SRNS (secondary)	ND	Weak Hypermetropia, Retinal Angiopathy
2706	ND	No	c.4055C>T	p. Ala1352Val	17 (HOM, ND)	*D. rerio*	5 / 245,932 (het)	33 yr	SSNS	ND	complete remission with Pred within 6 mo. Stayed in complete remission for 8 yrs. Only one reoccurrence since then.	FSGS (33 yr)	ND
CDK20													
A5013-21	Arab	Yes	c.610T>C	p.Phe204Leu	6 (HOM, M, P)	*C. elegans*	6 / 268,594 (het)	2.5 yr	SSNS	ND	complete remission by CsA; then remission by MMF without Pred (for 1.5 yrs)	MPGN (2.5 yr)	ND
ITSN1													
A3706<br>-21<br>-22	Arab	Yes	c.538C>T	p.Pro180Ser	7 (HOM, M, P)	*C. elegans*	NP	10 mo<br>3 mo	SRNS<br>SRNS, CNS	ND<br>ND	no response to Pred/CTX/CsA	MCNS (10 mo)<br>ND	ND<br>ND
A977-21	Arab	No	c.905A>G<br>c.4426A>G	p.Glu302Gly<br>p.Lys1476Glu	10 (het, ND)<br>36 (het, ND)	*C. elegans*<br>*C. intestinalis*	3 / 246,204 (het)<br>NP	3 yr	SSNS	ND	partial response to CTX (for long time)	MCNS (3 yr) & FSGS (9 yr)	ND
A2274-21	Europe	No	c.2554G>A<br>c.4550A>G	p.Asp852Asn<br>p.Lys1517Arg	18 (het, ND)<br>36 (het, ND)	*C. elegans*<br>*C. intestinalis*	5 / 229,410 (het)<br>NP	ND	SSNS	ND	partial response to CsA	ND	ND
ITSN2													
Pat<br>1<br>2	Japan	No	c.1016C>G<br>c.3640_3641	p.Ser339Cys<br>p.Ile1214Serfs*2	11 (het, M)<br>30 (het, P)	*D. melanogaster*<br>NA	8 / 243,070 (het)<br>5 / 241,902 (het)	2 yr<br>2 yr	SDNS<br>SDNS	ND<br>ND	complete remission by CsA<br>complete remission by CsA amd MZR	MCNS<br>MCNS	ND<br>ND
A3384	Arab	Yes	c.1313A>G	p.Glu438Gly	12 (HOM, M, P)	*C. elegans*	154 / 244,990 (het)<br>3 / 244,990 (HOM)	1.4 yr	SSNS	ND	complete remission with steroid combined with CTX then combined with CsA Frequent relapse	MPGN	ND

ACE-I, ACE inhibitor; *C. elegans*; Caenorhabditis elegans; *C. intestinalis*, Ciona intestinalis; CNS, congenital nephrotic syndrome; CsA, cyclosporine A; CTX, cyclophosphamide; *D. rerio,* Danio rerio; *D. melanogaster,* Drosophila melanogaster; DMS, diffuse mesangial sclerosis; ESKD, end stage kidney disease; ExAC, Exome Aggregation Consortium; FSGS, focal segmental glomerulosclerosis; *G. gallus,* Gallus gallus; het, heterozygous in affected individual; HOM, homozygous in affected individual; HT, hypertension; M, heterozygous mutation identified in mother; MCNS, minimal change nephrotic syndrome; mo, months; MMF, mycophenolate mofetil; MPGN, mesangioproliferative glomerulonephritis; MZR, mizoribine; NA, not applicable; ND, no data or DNA available; NP, not present; P, heterozygous mutation identified in father; Pred, prednisolone; RTX, resiniferatoxin; SDNS, steroid-dependent nephrotic syndrome; SRNS, steroid-resistant nephrotic syndrome; SSNS, steroid-sensitive nephrotic syndrome; wks, weeks; yr, years.

[a]A founder mutation from India is underlined.

Supplementary Table 2: Filtering process for variants from normal reference sequence following homozygosity mapping (HM) and whole exome sequencing (WES) in 3 families (A1358, A5013 and A3706) with NS.

FAMILY	A1358	A5013	A3706
[a]INDIVIDUALS SENT FOR WES	A1358-21	A5013-21	A3706-21
Consanguinity	Yes	Yes	Yes
Number of homozygosity peaks	[b]10	[b]12	[b]17
Cumulative homozygosity by descent [Mb]	196	242	106
[c]Hypothesis from mapping	Hom	Hom	Hom
Total sequence reads (Million)	56	78	81
Matched Reads	98.1%	98.7%	97.8%
Total number of variants detected	327,837	506,701	300,796
Reject common dbSNP137; MAF>1%; variants remaining	142,377	253,896	137,540
Keep VF>=55% AND cov >=2	71,383	155,483	82,102
Keep non-synonymous and splice	1,299	1,280	1,409
Keep located in hom peaks (Located within splice site; deletioninsertion; stop gained/stop lost; Missenses)	106	71	199
Remaining variants after inspection	8	1	16
[d]Sanger confirmation / segregation in family	1	1	1
Causative gene identified	***TNS2***	***CDK20***	***ITSN1***
Mutation effect on gene product	**Arg292Gln (Hom)**	**Phe204Leu (Hom)**	**Pro180Ser (Hom)**

[a] see Supplementary Table 1

[b] see Fig. 1

[c] evaluation for homozygous variants was done in regions of homozygosity by descent for affected individuals

[d] red numbers denote number of filtered-down variant(s) that contained the disease causing gene

cov, coverage; hom; homozygous; het, heterozygous; MAF, minor allele frequency; NA , not applicable; NS, Nephrotic Syndrome; VF, variant frequency

Supplementary Table 3: Filtering process for variants from normal reference sequence following whole exome sequencing (WES) in the Japanese family (Pat) with NS.

FAMILY	PAT				
**FAMILY MEMBERS**	**Pat1**	**Pat2**	**Father**	**Mother**	**Unaffected sibling**
Total sequence reads (Million)	172	174	191	147	181
Mapped reads	99.5%	99.5%	99.0%	99.5%	97.3%
Total number of variants detected	1,120,672	1,079,713	1,177,336	1,001,731	1,052,434
Filtering for low-quality variants	926,235	897,251	NA	NA	NA
Keep non-synonymous and splice	10,906	11,096	NA	NA	NA
Keep 1000genomes MAF<0.01	1,176	1,217	NA	NA	NA
Keep Esp6500I MAF<0.01	1,086	1,121	NA	NA	NA
Genes with hom or multiple variants	137 genes (454 variants)	139 genes (429 variants)	NA	NA	NA
Autosomal reccesive segregation	2 genes (*ITSN2*, *KIF19*)				
Absent homozygote in HGVD	1 gene (*ITSN2*)				
Causative gene identified	***ITSN2***				
Mutations effect on gene product	p.Ser339Cys (het)<br>p.Ile1214Serfs*2 (het)	p.Ser339Cys (het)<br>p.Ile1214Serfs*2 (het)	p.Ile1214Serfs*2 (het)	p.Ser339Cys (het)	p.Ile1214Serfs*2 (het)

Supplementary Table 4. Variant Frequencies (ExAC, GnomAD and in-house NPHP cohort), and *in-silico* prediction scores for all mutations identified in genes *MAGI2*, *TNS2*, *DLC1*, *CDK20, ITSN1* and *ITSN2* in NS families.

Family -Individual	Nucleotide change	Amino acid change	Exon (zygosity, segregation)	ExAC Allele count (zygosity)	GnomAD Allele count (zygosity)	In-house control cohort (248 NPHP exomes)	Mutation Taster[a]	Polyphen 2[b]
MAGI2								
A5146 -21	c.115G>T	p.Gly39*	1 (HOM, M, P)	NP	NP	NP	NA	NA
B91 (1302/11)	c.2238C>G	p.Tyr746*	12 (HOM, maternal isodisomy)	NP	NP	NP	NA	NA
TNS2								
A1358 -21	c.875G>A	p.Arg292Gln	11 (HOM, ND)	1 / 94,116 (het)	NP	NP	DC	0.995
A283 -21	c.1334G>A<br>c.3382A>G	p.Arg445Gln<br>p.Thr1128Ala	17 (het, M)<br>20 (het, P)	25 / 121,174 (het)<br>73 / 111,610 (het)	68 / 277,108 (het)<br>1522/ 240,618 (het)	NP<br>2 / 248 (het)	DC<br>Poly	0.003<br>0
2605	c.1675G>A	p. Gly559Arg	17 (HOM, ND)	10 / 50,270 (het)	34 / 160,586 (het)	NP	DC	0.967
A3775 -21	c.2574C>G	p.Ile858Met	18 (HOM, ND)	91 / 106,314 (het)	186 / 232,392 (het)	NP	Poly	0.206
A1640 -21	c.1693C>T<br>c.2574C>G	p.Arg565Trp<br>p.Ile858Met	18 (het, P)<br>18 (het, M)	14 / 41,686 (het)<br>91 / 106,314 (het)	22/ 119,318 (het)<br>186 / 232,392 (het)	NP<br>NP	DC<br>Poly	0.976<br>0.206
DLC1								
A548 -21	c.29G>A<br>c.128G>C	p.Trp10*<br>p.Ser43Thr	2 (het, ND)<br>2 (het, ND)	NP<br>1/ 121,402 (het)	NP<br>2/ 246,110 (het)	NP<br>NP	NA<br>Poly	NA<br>0.202
A4967 -21	c.539A>C<br>c.4073A>C	p.Glu180Ala<br>p.Lys1358Thr	2 (het, M)<br>15 (het, P)	NP<br>NP	NP<br>NP	NP<br>NP	Poly<br>DC	0.024<br>0.999
A3118 -21	c.1367C>T	p.Ala456Val	6 (HOM, M, P)	NP	NP	NP	DC	0.999
2706	c.4055C>T	p. Ala1352Val	17 (HOM, ND)	2 / 120,712 (het)	5 / 245,932 (het)	NP	DC	0.193

CDK20								
A5013<br>-21	c.610T>C	p.Phe204Leu	6<br>(HOM, M, P)	NP	6 / 268,594 (het)	NP	DC	0.991
ITSN1								
A3706<br>-21<br>-22	c.538C>T	p.Pro180Ser	7<br>(HOM, M, P)	NP	NP	NP	DC	0.968
A977<br>-21	c.905A>G<br>c.4426A>G	p.Glu302Gly<br>p.Lys1476Glu	10 (het, ND)<br>36 (het, ND)	2 / 122,860 (het)<br>NP	3 / 246,204 (het)<br>NP	NP<br>NP	DC<br>DC	0.462<br>0.967
A2274<br>-21	c.2554G>A<br>c.4550A>G	p.Asp852Asn<br>p.Lys1517Arg	18 (het, ND)<br>36 (het, ND)	3 / 115,790 (het)<br>NP	5 / 229,410 (het)<br>NP	NP	DC<br>DC	0.996<br>0.980
ITSN2								
Pat<br>1<br>2	c.1016C>G<br>c.3640_3641<br>del	p.Ser339Cys<br>p.Ile1214Serfs*2	11 (het, M)<br>30 (het, P)	8 / 120,760 (het)<br>5 / 121,366 (het)	8 / 243,070 (het)<br>5 / 241,902 (het)	NP<br>NP	DC<br>NA	0.354<br>NA
A3384	c.1313A>G	p.Glu438Gly	12<br>(HOM, M, P)	86 / 121284 (het)<br>2 / 121284 (HOM)	154 / 244,990 (het)<br>3 / 244,990 (HOM)	NP<br>NP	DC	0.994

DC, disease causing; ExAC, Exome Aggregation Consortium; GnomAD, the genome aggregation database; het, heterozygous in affected individual; HOM, homozygous in affected individual; M, heterozygous mutation identified in mother; NA, not applicable; ND, no data or DNA available; NP, not present; P, heterozygous mutation identified in father;

[a] http://www.mutationtaster.org/

[b] http://genetics.bwh.harvard.edu/pph2/

Supplementary Table 5: Effect of overexpression or knockdown of novel and known NS causing gene products on small G-proteins, cell migration and animal models.

Proteins of interest	active RhoA	active Rac1	active Cdc42	cell migration	Zebrafish model	KO Mouse Model
overexpression / knockdown	HEK293T	HEK293T	HEK293T or COS7	Human Podocytes / acute k.d. (siRNA)		
MAGI2	↔(m) ↑					
MAGI2 (knockdown)	↓ ↔(m)			↓		KO mice has NS [1-3]
TNS2	↔(m) ↑	↔	↔			
TNS2 (knockdown)	↓[4] ↓ ↔(m)	↔	↔	↔ ↑[4]		KO mice has NS [5]
DLC1	↓ ↔(m)	↔	↔			
DLC1 (knockdown)	a ↔[6] ↑	↑	↓	↓[6]		KO mice is E.L. [7]
CDK20	↔(m) ↑	↔	↑			
CDK20 (knockdown)	↓ ↔(m)	↔	↓	↓		
ITSN1	↔	↔	↔(m) ↑			
ITSN1 (knockdown)	↔	↔	↓			
ITSN2	↔	↔	↔(m) ↑			
ITSN2 (knockdown)	↔	↔	↓			KO mice has NS
DLC1 + TNS2	↔					
DLC1 + CDK20	↔					
DLC1 + MAGI2	↑					
CDK5				↓[6] ↓		
CDK5 (knockdown)						
CAV1					overexpression of cav1 in zebrafish podocytes is sufficient to induce NS which can be treated with glucocorticoids. [8]	
CAV1 (knockdown)	↓[10]	↔[9] ↑[10]	↑[1]	↓[10]		Cav1−/− mice showed a signifi -cantly slower wound healing rate compared with WT [10]
ARHGDIA						
ARHGDIA (knockdown)	↔	b ↑[11]	b ↑[1]	↑[1]	arhgdia-deficient zebrafish: RAC1 inhibitors were partially effective in ameliorating arhgdia-associated defects. The RhoA inhibitors showed no effect. [11]	
EMP2						
EMP2 (knockdown)		↓				
FAT1						
	↓[12]		↓[12]	↓[12]		Podocyte specific Fat1 deletion disrupts podocyte differentiation [12]
KANK						
KANK1 (knockdown)	↑[13]	↑[13]				
KANK2 (knockdown)	↑[13]			↓[13]	Knockdown of kank2 in zebrafish recapitulated a nephrotic syndrome phenotype, resulting in proteinuria and podocyte foot process effacement. [13]	

a *Data is based on pull-down by GST-RhoA and not G-LISA.*

b *Data is based on pull-down by GST-PAK1 and therefore the increase can be due to active Rac1 or Cdc42 or both.*

**Black** = Found/Validated in FH Lab
**Green** = Based on Literature
**Blue** = Based on hypothesis

Literature:

1 Balbas MD et al. MAGI-2 scaffold protein is critical for kidneybarrier function. ***Proc Natl Acad Sci USA*** (2014).
2 Ihara K et al. MAGI-2 is critical for the formation and maintenance of the glomerular filtration barrier in mouse kidney. ***Am J Pathol*** (2014).
3 Lefebvre J et al. Alternatively spliced isoforms of WT1 control podocyte-specific gene expression. ***Kidney Int*** (2015).
4 Clark K et al. Tensin 2 modulates cell contractility in 3D collagen gels throughthe RhoGAP DLC1. ***J Cell Biochem*** (2010).
5 Nishino T et al. The 129 genetic background affects susceptibility to glomerulosclerosis in tensin-2 deficient mice. ***Biomedical Research*** (2012).
6 Tripathi BK et al. CDK5 is a major regulator of the tumorsuppressor DLC1. ***J of Cell Biol*** (2015).
7 Durkin ME et al. DLC-1, a Rho GTPase-activating protein with tumor suppressorfunction, is essential for embryonic development. ***FEBS*** 2005.
8 Wan X et al. Loss of Epithelial Membrane Protein 2 AggravatesPodocyte Injury via Upregulation of Caveolin-1. ***JASN*** (2015).
9 Nethe M et al. Focal-adhesion targeting links caveolin-1 to a Rac1-degradation pathway. ***J Cell Science*** (2010)
10 Garcia AG et al. Caveolin-1 regulates cell polarization and directional migration through Src kinase and Rho GTPases. ***JCB*** (2007)
11 Gee YH et al. ARHGDIA mutations cause nephrotic syndrome via defective RHO GTPase signaling. ***J Clin Invest*** (2013)
12 Gee YH et al. FAT1 mutation cause a glomerulotubular nephropathy. ***Nat Commun*** (2016)
13 Gee YH et al. KANK deficiency leads to podocyte dysfunction and nephrotic syndrome. ***J Clin Invest*** (2015)

Supplementary Table 6. Mutations in *MAGI2*, *TNS2*, *DLC1*, *CDK20, ITSN1* and *ITSN2* in 17 families with NS interfere with multiple aspects of pathogenesis of NS.

Family -Individual	Nucleotide change	Amino acid change	Exon (Zygosity, Segregation)	Loss of function shown in: Active RhoA/Rac1/Cdc42	Loss of function shown in: Coimmunoprecipi-tation	Loss of function shown in: Cell migration	Loss of function shown in: Immunofluorescence (colocalization *or* filopodia induction)	KO Mouse Model
MAGI2								
A5146 -21	c.115G>T	p.Gly39*	1 (HOM, M, P)	Yes (RhoA)	Yes (abrogation of interaction with TNS2 and DLC1)	*no readout*	Yes (Mislocalization with β-catenin at adherens junctions)	KO Mice have NS (Published) 1-3
B91 (1302/11)	c.2238C>G	p.Tyr746*	12 (HOM, maternal isodisomy)	Yes (RhoA)	Yes (abrogation of interaction with TNS2)	*no readout*	Yes (Mislocalization with β-catenin at adherens junctions)	KO Mice have NS (Published) 1-3
TNS2								
A1358 -21	c.875G>A	p.Arg292Gln	13 (HOM, ND)	Yes (RhoA)	No	*no readout*	-	KO Mice has NS (Published) 4
A283 -21	c.1334G>A<br>c.3382A>G	p.Arg445Gln<br>p.Thr1128Ala	17 (het, M)<br>20 (het, P)	Yes (RhoA)<br>Yes (RhoA)	No<br>No	*no readout*<br>*no readout*	-<br>-	KO Mice has NS (Published) 4
2605	c.1675G>A	p. Gly559Arg	17 (HOM, ND)	Yes (RhoA)	No	*no readout*	-	KO Mice has NS (Published) 4
A3775 -21	c.2574C>G	p.Ile858Met	18 (HOM, ND)	Yes (RhoA)	No	*no readout*	-	KO Mice has NS (Published) 4
A1640 -21	c.1693C>T<br>c.2574C>G	p.Arg565Trp<br>p.Ile858Met	18 (het, P)<br>18 (het, M)	Yes (RhoA)<br>Yes (RhoA)	No<br>No	*no readout*<br>*no readout*	-<br>-	KO Mice has NS (Published) 4
DLC1								
A548 -21	c.29G>A<br>c.128G>C	p.Trp10*<br>p.Ser43Thr	2 (het, ND)<br>2 (het, ND)	Yes (RhoA)<br>Yes (RhoA)	Yes (abrogation of interaction with CAV1)<br>No	Yes<br>No	-<br>-	-
A4967 -21	c.539A>C<br>c.4073A>C	p.Glu180Ala<br>p.Lys1358Thr	2 (het, M)<br>15 (het, P)	Yes (RhoA)<br>Yes (RhoA)	No<br>Yes (abrogation of interaction with CAV1)	No<br>Yes	-<br>-	-

**A3118<br>-21**	**c.1367C>T**	**p.Ala456Val**	**6<br>(HOM, M, P)**	**Yes (RhoA)**	**No**	**Yes**	**-**	**-**
**2706**	**c.4055C>T**	**p. Ala1352Val**	**17<br>(HOM, ND)**	**Yes (RhoA)**	**No**	**Yes**	**-**	**-**
CDK20								
**A5013<br>-21**	**c.610T>C**	**p.Phe204Leu**	**6<br>(HOM, M, P)**	**Yes (RhoA)**	**No**	***no readout***	**-**	**-**
ITSN1								
**A3706<br>-21<br>-22**	**c.538C>T**	**p.Pro180Ser**	**7<br>(HOM, M, P)**	**Yes (Cdc42)**	**-**	***no readout***	**Yes (decreased filopodia induction)**	**KO Mice show neuronal defects (Published) 5**
**A977<br>-21**	**c.905A>G<br><br>c.4426A>G**	**p.Glu302Gly<br><br>p.Lys1476Glu**	**10 (het, ND)<br><br>36 (het, ND)**	**Yes (Cdc42)<br><br>Yes (Cdc42)**	**-<br>-**	***no readout<br>no readout***	**Yes (decreased filopodia induction)<br>Yes (decreased filopodia induction)**	**KO Mice show neuronal defects (Published) 5**
**A2274<br>-21**	**c.2554G>A<br><br>c.4550A>G**	**p.Asp852Asn<br><br>p.Lys1517Arg**	**18 (het, ND)<br><br>36 (het, ND)**	**Yes (Cdc42)<br><br>Yes (Cdc42)**	**-<br>-**	***no readout<br><br>no readout***	**Yes (decreased filopodia induction)<br>Yes (decreased filopodia induction)**	**KO Mice show neuronal defects (Published) 5**
ITSN2								
**Pat<br>1<br>2**	**c.1016C>G<br><br>c.3640_3641 del**	**p.Ser339Cys<br><br>p.Ile1214Serfs*2**	**11 (het, M)<br><br>30 (het, P)**	**Yes (Cdc42)<br><br>Yes (Cdc42)**	**-<br><br>-**	***no readout<br><br>no readout***	**Yes (decreased filopodia induction)<br>Yes (decreased filopodia induction)**	**$Itsn2^{-/-}$ mice develop incompletely penetrant NS upon LPS injection**
**A3384**	**c.1313A>G**	**p.Glu438Gly**	**12<br>(HOM, M, P)**	**Yes (Cdc42)**	**-**	***no readout***	**Yes (decreased filopodia induction)**	**$Itsn2^{-/-}$ mice develop incompletely penetrant NS upon LPS injection**

1. Balbas MD *et al.* MAGI-2 scaffold protein is critical for kidney barrier function. *Proc Natl Acad Sci USA* (2014).
2. Ihara K *et al.* MAGI-2 is critical for the formation and maintenance of the glomerular filtration barrier in mouse kidney. *Am J Pathol* (2014).
3. Lefebvre J *et al.* Alternatively spliced isoforms of WT1 control podocyte-specific gene expression. *Kidney Int* (2015).
4. Nishino T *et al.* The 129 genetic background affects susceptibility to glomerulosclerosis in tensin-2 deficient mice. *Biomedical Research* (2012).
5. Yu Y *et al.* Mice deficient for the chromosome 21 ortholog Itsn1 exhibit vesicle-trafficking abnormalities. *Hum Mol Genet* (2008).

het, heterozygous in affected individual; HOM, homozygous in affected individual; M, heterozygous mutation identified in mother; NA, not applicable; ND, no data or DNA available; P, heterozygous mutation identified in father.

Supplementary Table 7: Genes included in the targeted next generation sequencing.

Gene	Regions	Coverage
ITSN2	45	99.50%
ACTN4	25	100%
ACTR2	12	99.61%
ACTR3	12	98.62%
ARHGAP33	22	99.92%
ARHGDIA	4	100%
ARHGEF1	31	99.58%
ARHGEF12	39	99.70%
ARHGEF6	26	100%
ARHGEF7	31	99.94%
CDC42	10	97.41%
FGD1	17	100%
FGD3	21	99.05%
ITSN1	49	99.64%
MCF2L	48	99.81%
MYO1E	31	100%
PAK1	24	97.30%
PAK2	15	96.59%
RAC1	9	100%
RHOA	10	100%
WAS	14	100%

Supplementary Table 8: RNAi target sequences.

Primer	Target sequence
Human *MAGI2*, siRNA #7	GAGUCUGGAUCCACUAUAA
Human *TNS2*, siRNA #5	CUACACAUCUGGAGUCUAU
Human *DLC1*, siRNA #5	GCCGATGTCGTAATTCCTATA
Mouse *Dlc1*, siRNA #1	GGAUCAAGGUUCCAGACUA
Human *CDK20*, siRNA #9	AGGCACAGGUCAAGAGCUA
Human *ITSN1*, siRNA #6	GAACGAAAGAUCAUAGAAU
Human *TNS2* shRNA #1	gatccGCATGACCTGACCCGCTTAAACGAATTTAAGCGGGTCAGGTCATGCTTTTTTACGCGTg
	aattcACGCGTAAAAAAGCATGACCTGACCCGCTTAAATTCGTTTAAGCGGGTCAGGTCATGCg
Human *DLC1* shRNA #2	gatccgcacaaacagaggactgccattattcgaaaataatggcagtcctctgtttgtgcTTTTTTACGCGTg
	aattcACGCGTAAAAAAgcacaaacagaggactgccattattttcgaataatggcagtcctctgtttgtgcg
